# Epistatic Module Detection for Case-Control Studies: A Bayesian Model with a Gibbs Sampling Strategy

**DOI:** 10.1371/journal.pgen.1000464

**Published:** 2009-05-01

**Authors:** Wanwan Tang, Xuebing Wu, Rui Jiang, Yanda Li

**Affiliations:** 1 MOE Key Laboratory of Bioinformatics and Bioinformatics Division, TNLIST and Department of Automation, Tsinghua University, Beijing, China; University of California San Diego and The Scripps Research Institute, United States of America

## Abstract

The detection of epistatic interactive effects of multiple genetic variants on the susceptibility of human complex diseases is a great challenge in genome-wide association studies (GWAS). Although methods have been proposed to identify such interactions, the lack of an explicit definition of epistatic effects, together with computational difficulties, makes the development of new methods indispensable. In this paper, we introduce epistatic modules to describe epistatic interactive effects of multiple loci on diseases. On the basis of this notion, we put forward a Bayesian marker partition model to explain observed case-control data, and we develop a Gibbs sampling strategy to facilitate the detection of epistatic modules. Comparisons of the proposed approach with three existing methods on seven simulated disease models demonstrate the superior performance of our approach. When applied to a genome-wide case-control data set for Age-related Macular Degeneration (AMD), the proposed approach successfully identifies two known susceptible loci and suggests that a combination of two other loci—one in the gene *SGCD* and the other in *SCAPER*—is associated with the disease. Further functional analysis supports the speculation that the interaction of these two genetic variants may be responsible for the susceptibility of AMD. When applied to a genome-wide case-control data set for Parkinson's disease, the proposed method identifies seven suspicious loci that may contribute independently to the disease.

## Author Summary

Although genome-wide association studies (GWAS) have been quite popular due to recent advances in low-cost genotyping techniques, most of the reported studies only analyze single-locus effects because traditional multi-locus methods are not computationally practical in the detection of epistatic interactive effects of multiple loci. Here, on the basis of a rigorous definition of epistatic modules that describe interactive effects of multiple loci, we take advantage of a Bayesian model with a properly designed Gibbs sampling strategy to facilitate the detection of such modules. We confirm via extensive simulation studies that the proposed method, named *epi*MODE, is not only feasible in detecting multi-locus effects but also more powerful than three representative methods on seven disease models. We apply the proposed method to an Age-related Macular Degeneration (AMD) data and discover that a combination of two loci—one in the gene *SGCD* and the other in *SCAPER*—might be associated with AMD. Considering its advantages, we suggest that the proposed method be applied to more GWAS data for the detection of multi-locus interactive effects.

## Introduction

With the development of modern human and medical genetics, it has been widely accepted that genetic variation plays an important role in the pathogenesis of genetic inherited diseases [1]. The identification of causative genetic variants therefore becomes the primary step towards the understanding of genetic principles underlying these diseases. For Mendelian diseases in which an individual genetic variant in a single gene is both sufficient and necessary to cause a disease, classical statistical approaches such as linkage analysis [2]–[5] and association studies [6],[7] have shown remarkable successes in the identification of causative genetic variants. Nevertheless most common diseases are complex ones that are supposed to be caused by multiple genetic variants, their interactive effects, and/or their interaction with environment factors [7],[8]. The detection of such interactive effects therefore plays a key role in the understanding of these diseases.

The interactive effects of multiple genetic variants underlying complex diseases are often referred to as epistasis or epistatic interactions. Recent advances in biomedical studies have been confirming the contribution of epistasis to complex diseases. For example, Tiret *et al* reported synergistic effects of polymorphisms in the angiotensin-converting enzyme and the angiotensin-II type 1 receptor gene on the risk of myocardial infarction [9]. Ritchie *et al* identified the association of a high-order interaction among four polymorphisms in three estrogen-metabolism genes with breast cancer [10]. Williams *et al* reported the influence of a two-locus interaction between polymorphisms in the angiotensin converting enzyme and the G protein-coupled receptor kinase on hypertension susceptibility [11]. Tsai *et al* identified the association of a three-locus interaction among polymorphisms in renin-angiotensin system genes with atrial fibrillation [12]. Cho *et al* reported the association of a two-locus interaction between polymorphisms in the uncoupling protein 2 gene and the peroxisome proliferator-activated receptor gamma gene with Type 2 diabetes mellitus [13]. Martin *et al* reported the influence of a two-locus interaction between polymorphisms in KIR3DL1 and HLA-B on both AIDS progression and plasma HIV RNA [14]. With these examples, epistasis between multiple genetic variants is now widely believed to be the causative pattern of human complex diseases.

In order to detect epistasis, a number of multi-locus approaches have been developed. For example, Hoh *et al* proposed a trimming, weighting, and grouping approach that used the summation of statistics on the basis of single-locus marginal effects and the Hardy-Weinberg equilibrium (HWE) for hypothesis testing [15]. Nelson *et al* proposed a combinatorial partitioning method (CPM) that exhaustively searched for a combinatory genotype group that had the most significant difference in the mean of the responding continuous phenotype [16]. Culverhouse *et al* proposed a restricted partitioning method (RPM) which modified CPM by ignoring partitions that combined individual genotypes with very different mean trait values [17]. Millstein *et al* proposed a focused interaction testing framework (FITF) in which a prescreening strategy was developed to reduce the number of tests [18]. Chatterjee *et al* used Turkey's 1-degree-of-freedom model to detect interacting loci from different regions [19]. Ritchie *et al* proposed a multifactor-dimensionality reduction (MDR) method in which exhaustive search was performed to detect combinations of loci with the highest classification capability [10].

Although these methods have shown their successes in association studies for small scale candidate genes [10], [15]–[19], their effectiveness for large scale case-control data has not yet been validated. Besides, most of the methods rely strongly on exhaustive search for combinations of multiple loci. This search strategy, though feasible when the number of candidate genetic variants is small, can hardly be computationally practical for large scale or whole-genome association studies in which the number of candidate genetic variants is typically very huge. For example, a study on Age-related Macular Degeneration (AMD) has genotyped more than 100 thousand single nucleotide polymorphism (SNP) markers for 96 patients and 50 unaffected people [20], and a recent genome-wide association study on Parkinson's disease has genotyped more than 400 thousand SNP markers for 270 patients and 271 unaffected people [21],[22]. With such dense SNPs being genotyped, methods based on exhaustive search are computationally impractical due to the vast number of possible combinations of the SNP markers. The main challenge for genome-wide association studies is therefore to design computational approaches that are capable of avoiding the “combinatorial explosion” curse to identify epistatic interactions.

A recent breakthrough in genome-wide epistasis mapping is the introduction of the Bayesian epistasis association mapping (BEAM) method [23] that integrates a Bayesian model with the Metropolis-Hastings algorithm to infer the probability that each locus is associated with the susceptibility of a specified disease. BEAM classifies SNP markers into three types: SNPs unassociated with the disease, SNPs contributing to the disease susceptibility independently, and SNPs influencing the disease risk jointly with each other. However, the genetic models for complex diseases could be far more complicated than that proposed by BEAM. For example, the disease-associated SNPs that jointly influence the disease risk may be further divided into subgroups, in which a SNP interacts with other SNPs in the same subgroup, but not with those in the other subgroups. This situation could be very common in real data, making BEAM ineffective in the exploration of true interactive effects of multiple loci.

To overcome this limitation, in this paper, we give an explicit presentation of “epistasis” and define “epistatic modules” as basic units of disease susceptibility loci. On the basis of this notion, we put forward a Bayesian marker partition model to explain the observed case-control data and further generalize this model to account for the existence of linkage disequilibrium (LD) between genetic variants. To facilitate the identification of epistatic modules, we develop a Gibbs sampling strategy with a reversible jump Markov chain Monte Carlo (RJ-MCMC) procedure to simulate the posterior distribution that genetic variants belong to the epistatic modules and further resort to hypothesis testing to screen out statistically significant modules. In contrast to most of the existing methods that entirely or partially rely on exhaustive search for combinations of loci, the proposed approach, named *epi*MODE (*epi*static MOdule DEtection), natively identifies interactive loci (epistatic modules) without enumerating their combinations, thereby being capable of detecting interactive effects of multiple loci from a vast number of genotyped genetic variants.

We systematically compare the proposed approach with three existing methods on seven simulated disease models. The results show the superior performance of our approach over the other methods. We further apply the proposed approach to a genome-wide case-control data set for Age-related Macular Degeneration (AMD) that contains more than 100 thousand SNPs genotyped for 96 cases and 50 controls [20] and successfully identify two SNPs that are known to be associated with the disease. Besides, the results also suggest that two other SNPs (rs1394608 and rs3743175) may have interactive effects on the susceptibility of the disease. We also apply the proposed approach to a genome-wide case-control data set for Parkinson's disease (400 thousand SNPs genotyped for 270 cases and 271 controls) [21],[22] and identify seven SNP markers that may be associated with the disease.

## Materials and Methods

### Epistasis and Epistatic Modules

The concept of epistasis implies that the phenotypic effect of one locus is dependent on one or more other loci. Nonetheless the definitions of epistasis in biology and statistics are not exactly consistent. Even from the statistical perspective only, researchers have different understandings of epistasis [24],[25]. Considering these inconsistencies, it is necessary to first give a clear definition of epistasis, for the purpose of developing a computational method for identifying multiple loci that contribute to the disease susceptibility.

In this paper, a locus stands for a SNP. A genotype stands for a set of two alleles (one inherited from father and the other from mother) at a locus and has three possible values: homozygosity of common alleles, homozygosity of minor alleles, and heterozygosity. A combinatory genotype represents the genotype of a combination of multiple loci. For a combination of *t* loci, the number of all possible combinatory genotypes is 3*^t^*. The penetrance of a combinatory genotype is the probability/risk that an individual with this combinatory genotype is affected, given the combinatory genotype of the multiple loci. We first assume that all loci are in linkage equilibrium, also known as independent, and then we generalize the definitions to the situation with linkage disequilibrium between multiple loci.

Let 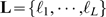 be the set of all *L* loci under investigation, and 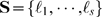 be the set of all *s* disease susceptibility loci that determine the disease risk. For any two subsets, **S**_1_ and **S**_2_, of **S** (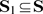, 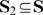, and 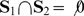), their penetrance given the combinatory genotypes 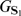 and 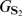 respectively, can be described as
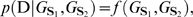
where 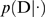 represents the penetrance of a given combinatory genotype, 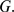 a combinatory genotype of the multiple loci, and 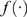 the function denoting how combinatory genotypes determine the disease penetrance.

For any given combinatory genotypes of **S**_1_ and **S**_2_, if
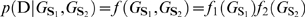
is always true, the relationship between the two subsets of loci **S**_1_ and **S**_2_ is defined as “independently contributing” to the disease. Otherwise, the relationship between **S**_1_ and **S**_2_ is defined as “epistasis.” Particularly, the relationship between a set of loci and a null set is defined as epistasis.

A set of loci 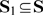 is an “epistatic module” if and only if the relationship between **S**_1_ and its complement, 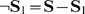, is “independently contributing,” that is, for any given genotype 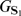 and 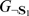,
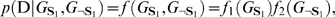
and the relationship between any subset of **S**_1_, 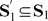, and its complement 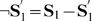 is epistasis.

Obviously, the set of disease susceptibility loci **S** consists of one or more epistatic modules. We further verify that there is no overlap between any two epistatic modules, and epistatic modules are independent in both case and control populations (Text S1).

In genome-wide association studies where the SNPs are quite dense, it is common that a SNP may be in LD with other SNPs. To account for this situation, we define a group of SNPs that are in LD with each other as an “LD set” and extend the above definition of epistatic modules by replacing individual loci with LD sets. Note that with this extension, all properties of epistatic modules remain unchanged, as long as we treat an LD set as an individual locus in the previous derivation.

**Figure 1:**
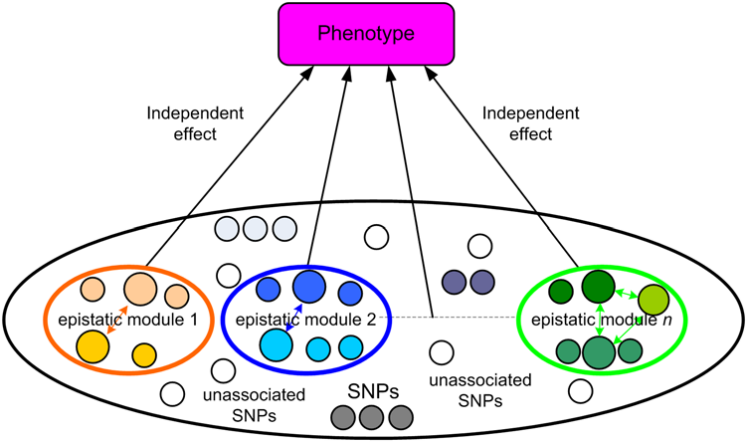
Relationship between phenotype and genotype, illustrated with epistatic modules. Disease-associated SNPs are contained in epistatic modules. Disease-unassociated SNPs are outside the modules. SNPs with the same color form an LD set.

The mechanism how a number of susceptibility SNPs contribute to the disease risk through epistatic modules is shown in Figure 1. The disease risk is determined by a number of epistatic modules, each of which contributes to the disease independent of the others. An epistatic module is composed of one or more susceptibility SNPs, each of which may be in LD with some other SNPs, forming an LD set. A disease susceptibility SNP, together with the SNPs that are in LD with it, relies on other disease susceptibility SNPs or LD sets in the same epistatic module to affect the disease susceptibility. An epistatic module cannot be further divided into smaller epistatic modules; hence epistatic modules are the smallest genetic units that independently influence the disease risk.

### Bayesian Marker Partition Model

Suppose that in a population-based case-control study, 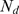 cases and 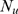 controls are genotyped at a number of *L* SNP markers. The genotypes for cases and controls are represented as 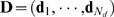 and 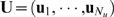, respectively, where 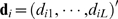 and 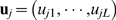 denote the genotypes of the *i*-th patient and the *j*-th unaffected individual at the *L* markers, respectively. With the understanding of epistatic modules, the *L* markers can be partitioned into 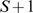 modules *M*_0_, *M*_1_,…, *M_S_*, with *M*_0_ containing markers unlinked to the disease and *M*_1_ to *M_S_* being epistatic modules.

Let 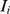 (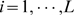) be an indicator of the assignment of the *i*-th marker into one of the 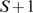 modules, and 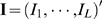 be a vector representing the assignments for all of the *L* markers. Obviously, 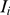 has 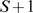 possible values 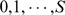. Let *l_m_* be the number of markers falling into the *m*-th module (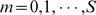). We have that 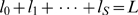. Let **D** *_m_* and **U** *_m_* be the genotypes of the sets of markers that belong to the *m*-th module in the case and the control populations, respectively. Obviously, we have that 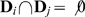 when 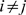 and 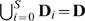 for the case population, and 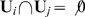 when 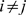 and 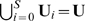 for the control population.

With these concepts, the problem of finding markers that have epistatic interactions on the disease risk is equivalent to a problem of assigning the markers to epistatic modules. Particularly, the assignment for a marker can be done by first calculating the probability of the observed data given a certain marker partition pattern and then obtaining the posterior probability that the marker belongs to each module using some sampling strategy. For a clear presentation, we first derive a Bayesian model that assumes independence between SNPs and then generalize the model to account for the existence of LD sets.

The module *M*_0_ consists of markers that are unlinked to the disease. Therefore, markers in **D**_0_ (the case population) should follow the same distribution as those in **U**_0_ (the control population). Let 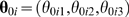, 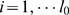, be the probabilities of occurrence of the three possible genotypes for the *i*-th marker in *M*_0_, and 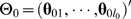 be the vector that is composed of all probabilities of genotypes of the *l*_0_ markers belonging to *M*_0_. Let 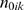 and 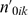 be the number of individuals that have the *k*-th genotype at the *i*-th marker in the case and the control populations, respectively. The joint distribution of the observed genotypes **D**_0_ and **U**_0_, given the partition **I** and the parameters 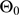 can then be written as
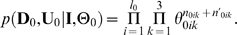
(1)


Following the Bayesian approach, we assume that every 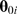 (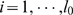) follows a Dirichlet distribution with the hyper-parameter 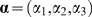, that is, 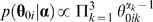. Integrating out 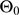 in Equation (1), we obtain
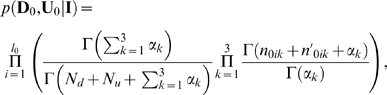
(2)where 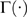 is the Gamma function.

For an epistatic module *M_m_* (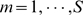) containing *l_m_* SNPs, there are a total of 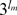 combinatory genotypes. Let 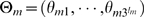 and 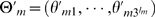 be the probabilities of occurrences of all combinatory genotypes in the case and the control populations, respectively. Let 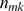 and 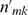 be the numbers of occurrences of the *k*-th combinatory genotype in the case and the control populations, respectively. The distributions of **D** *_m_* and **U** *_m_*, given the parameters 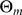 and 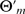, can be written as
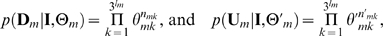
respectively.

Assuming that 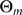 and 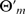 follow Dirichlet prior distributions with hyper-parameters 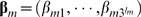 and 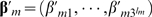, respectively, we integrate out 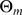 and 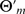 and obtain
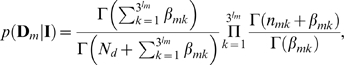
(3)and
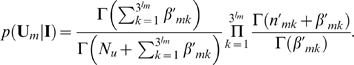
(4)As the distributions of **D** *_m_* and **U** *_m_* are independent, we have
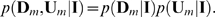
Putting the above likelihood functions together, we have the posterior distribution of **I**, given the observed genotypes, as
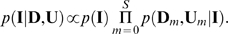



The prior distribution 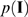 need to be determined in advance. For simplicity, we assume that the partition of the loci are independent, and for each locus, without prior knowledge, the probability that it belongs to the *m*-th module is 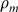 (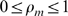 and 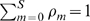). With these two assumptions, we have 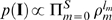. Note that when prior knowledge that can be used to infer the relationship between a locus and the disease risk is available, the corresponding 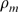 could be updated accordingly. We assume that all Dirichlet hyper-parameters are equal to 0.5 unless otherwise specified.

### Accounting for LD between Disease Susceptibility SNPs

We use a first-order Markov model to account for the situation in which a set of SNPs are in LD with a disease susceptibility SNP in an epistatic module, say, an LD set. For a clear presentation, we refer to the disease susceptibility SNP as the core SNP and SNPs in LD with it as peripheral SNPs.

Given a core SNP, the likelihood of the genotypes of a peripheral SNP in the case population is
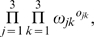
where 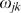 is the probability that the peripheral SNP has the *k*-th genotype conditional on that the core SNP has the *j*-th genotype, and 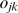 is the number of cases for which the core and peripheral SNPs have the *j*-th and *k*-th genotypes, respectively.

Assuming Dirichlet priors with hyper-parameters 
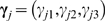
 for 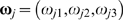, we integrate out 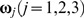 and obtain the posterior distribution of the genotypes of the peripheral SNP in the case population conditional on the core SNP as
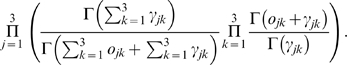



Suppose that in a module 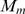 with 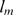 SNPs, there are 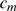 core SNPs and 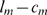 peripheral SNPs (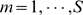). Let 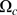 be the set of peripheral SNPs that are in LD with the *c*-th core SNP (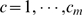). We have that the intersection of any two of these sets is empty, while the union of all these sets contains all peripheral SNPs. The posterior distribution of the genotypes of the set of peripheral SNPs 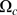 in the case population conditional on the *c*-th core SNP is given by
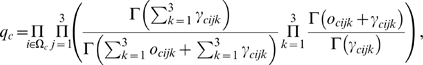
(5)where (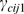, 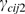, 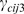) are Dirichlet hyper-parameters, and 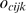 is the number of cases for which the *c*-th core SNP has the *j*-th genotype, and the *i*-th peripheral SNP has the *k*-th genotypes. Putting Equations (3) and (5) together, the likelihood of the genotypes in the case population 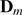 is
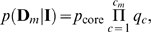
where *p*_core_ is given by Equation (3) as
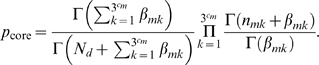



Similarly, by replacing the case population with the control population, the likelihood of the genotypes in the control population 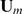 can be obtained as 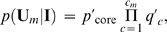
where 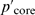 is given by Equation (4) as
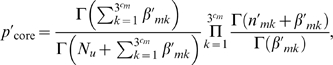
and 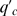 is given by
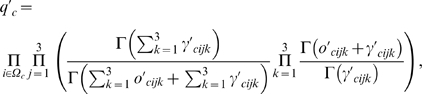
(6)where (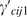, 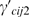, 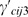) are Dirichlet hyper-parameters, and 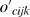 is the number of controls for which the *c*-th core SNP has the *j*-th genotype, and the *i*-th peripheral SNP has the *k*-th genotypes.

Finally, the likelihood of observing both the case and the control populations is given by
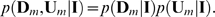
(7)We also assume that all Dirichlet hyper-parameters are equal to 0.5 unless otherwise specified.

### Accounting for LD between Disease Unassociated SNPs

The Bayesian marker partition model described above assumes independence between SNPs that are unlinked to the disease. Nevertheless the existence of LD may make distributions of genotypes of these SNPs dependent. In the model discussed above, there is no specific module for these linked disease-unassociated SNPs. As a result, these SNPs could be partitioned into some epistatic modules and negatively affect the correct partition of these modules. We therefore propose the use of LD modules to account for the existence of LD between disease-unassociated SNPs.

Although the distributions of genotypes for markers in LD are dependent in both the case and the control populations, as those for markers in epistatic modules, the underlying principle between LD markers and epistatic modules are quite different. For LD markers, the distributions of genotypes are almost the same for the case and the control populations, while for epistatic modules the distributions of genotypes are different between the case and the control populations. In order to incorporate this understanding into the Bayesian partition model, we assume that other than the *S* epistatic modules, there further exist *T* LD modules, labeled by {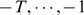}, in each of which loci are in strong LD with each other.

We also use a first-order Markov model to account for LD between the SNPs in an LD module. For an LD module 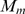 (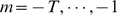), we assume that there exists a core SNP *c*, and the distributions of genotypes of all other (peripheral) SNPs in this LD module depend on the genotype of this core SNP. Let 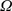 be the set of the 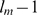 peripheral SNPs that are in LD with the core SNP. Using similar reasoning as for the epistatic modules, we obtain that
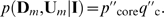


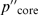
 is derived with a similar way as Equation (2) and is given by
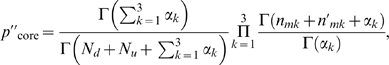
where 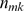 and 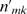 are the numbers of individuals that have the *k*-th genotype at the core SNP in the case and the control populations, respectively. 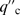 is derived with a similar way as Equation (6) and is given by
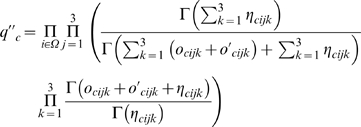
where 
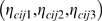
 are Dirichlet hyper-parameters, and 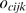 and 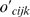 are the numbers of individuals for which the core SNP has the *j*-th genotype, and the *i*-th peripheral SNP has the *k*-th genotypes in the case and the control populations, respectively. We also assume that all hyper-parameters are equal to 0.5 unless otherwise specified.

With LD modules being incorporated, the posterior distribution for the generalized indicator vector 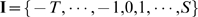 under the generalized Bayesian model is then
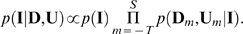



### Gibbs Sampling Strategy for Marker Partitioning

The posterior distribution of the partition **I** given by the above Bayesian partition model suggests the following Gibbs sampler
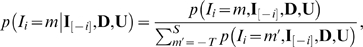
(8)where 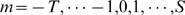 and 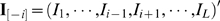. In order to calculate this sampler in an efficient way, we compute
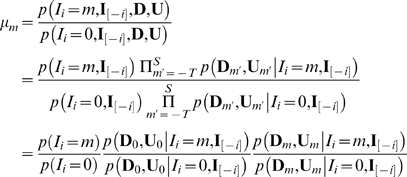
for 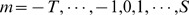, and then obtain
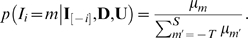
With this sampler, a Gibbs sampling algorithm can be performed as follow.

Step 1: Initialization. Assign module labels to indicators 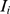 for 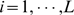, according to prior probabilities 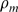 (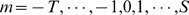).
Step 2: Gibbs sampling. Select an indicator 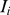 (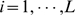) at random and update its module label according to the posterior probabilities 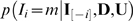 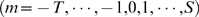.
Step 3: Repeat the above sampling iteration until convergence or a pre-defined maximum number of iterations being reached.

In order to calculate the Gibbs sampler, i.e., Equation (8), we need to partition SNPs in epistatic and LD modules into core SNPs and peripheral SNPs, say, to obtain structures of the modules. Besides, the numbers of modules (*S* and *T*) are also unknown. We will address these two questions in the following two sections.

### Obtaining Module Structures

Given a set of SNPs in an epistatic module, we need to partition the SNPs into non-overlap LD sets. For each LD set, we need to assign a core SNP. The partition of LD sets, together with the assignment of a core SNP for each LD set, is referred to as the structure of an epistatic module.

A naïve method for obtaining the structure of a module is to exhaustively search for all possible structures of the module and then select the one with the maximum likelihood. Specifically, for an epistatic module *M_m_* (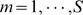) containing 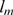 SNPs, there are 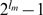 ways for selecting the core SNPs, corresponding to the different ways of selecting non-empty subsets from the 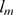 SNPs. Furthermore, in the case that the number of core SNPs is 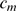, the number of ways for associating the rest 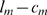 peripheral SNPs to the core SNPs is 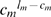, since each peripheral SNP can be assigned to one of the core SNPs, and the assignments are mutually independent. Obviously, the number of all possible structures of an epistatic module grows rapidly, making the exhaustive search strategy practical only when the module contains a small number of SNPs. We therefore propose the following sampling approach to search for a reasonable module structure when the exhaustive search strategy is hard to apply.

For an epistatic module 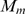 with 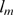 SNPs, in which 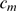 are core SNPs, and the rest 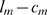 are peripheral ones, we index the core SNPs by numbers from 1 to 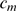, and we index the peripheral SNPs by numbers from 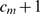 to 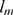. We further introduce an indicator vector 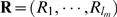, representing the status of all SNPs in the module. In this vector, 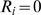 (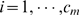) means that the *i*-th SNP is a core SNP, and 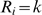 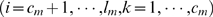 means that the *i*-th SNP is a peripheral SNP of the *k*-th core SNP.

Consider a peripheral SNP indexed by *i* (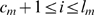). The posterior distribution of the indicator 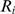, given the rest of the indicators 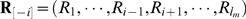 and the observation 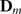 and 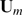, can be written as
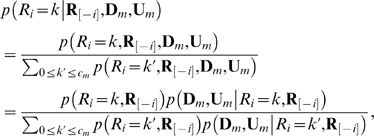
where the likelihood function can be calculated in a similar way as Equation (7). Assuming equal prior probabilities for all possible structures of the module, the above posterior distribution suggests the following Gibbs sampler for the peripheral SNP,
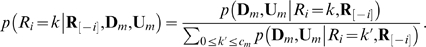
(9)


Consider a core SNP indexed by *i* (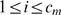). There are two situations: (1) the core SNP has some peripheral SNPs, and (2) the core SNP has no peripheral SNPs. In the former case, we need to fix the indicator 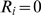. In the latter case, a Gibbs sampler can be obtained as
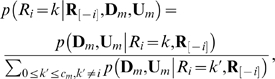
(10)where we exclude the situation in which the core SNP becomes its own peripheral SNP.

The above Gibbs samplers suggest the following sampling strategy:

Step 1: Initialization. Generate a random structure.
Step 2: Sampling. Select a SNP at random. If it is a peripheral SNP, sample its indicator according to Equation (9); if it is a core SNP with no peripheral SNPs, sample its indicator according to Equation (10); otherwise keep its indicator unchanged. After sampling, update the indices and indicators of the SNPs.
Step 3: Repeat the above sampling step until convergence or a pre-defined maximum number of iterations being reached to obtain the posterior distribution of module structures, and then sample a module structure according to this distribution.

To further reduce the computational burden, we propose the following forward and backward strategies that are very economy in terms of computation time.

In the forward strategy, we consider three situations of adding a SNP into an existing epistatic module. First, the SNP is itself a core SNP, and there are no other SNPs in LD with it. Second, the SNP is in LD with an existing core SNP, and this core SNP remains unchanged. Third, the SNP is in LD with an existing core SNP, but this core SNP needs to be updated as the added SNP. To deal with the first case, we try to create a new LD set to include the new SNP as the core SNP in constant time complexity. To deal with the second case, we try to add the new SNP as a peripheral SNP to every existing LD set in linear time complexity, proportional to the number of existing LD sets. To deal with the third case, we try to add the new SNP as the core SNP and downgrade the previous core SNP to a peripheral SNP for every existing LD set in linear time complexity, also proportional to the number of existing LD sets. Finally, we compare likelihood values of resulting structures of the above efforts and select the structure with the highest likelihood as the new module structure.

In the backward strategy, we also consider three situation of removing a SNP from an existing epistatic module. First, the SNP is in LD with a core SNP. Second, the SNP is itself a core SNP with no other SNPs in LD with it. Third, the SNP is a core SNP with some other SNPs in LD with it. The first and second cases can be dealt with in constant time complexity. The third case can be exhaustively searched for the new core SNP in linear time complexity, proportional to the number of SNPs in LD with the removed SNP. By comparing the likelihood values of these three cases, we can obtain a new structure for the module.

The exhaustive search strategy can provide optimal module structures, but its computation time is acceptable only when a module contains a small number of SNPs. The sampling strategy takes uncertainty in the partitioning process into consideration and can alleviate the computational burden when a module contains a large number of SNPs. The forward and the backward strategies can greatly reduce the computational burden and offer sub-optimal module structures. To achieve a reasonable trade-off between the computational burden and the optimality of module structures, we also propose a hybrid strategy in which we mainly perform the forward and the backward strategies and periodically apply the exhaustive search or the sampling methods. According to our experience, the hybrid strategy is much faster than the exhaustive search and the sampling methods and can yield similar results as the other two methods in most cases. Therefore, we suggest the use of the hybrid strategy.

Similar to epistatic modules, we need to also assign a core SNP for each LD module. However, the situation is quite simple for obtaining structures for LD modules, because an LD module has only one core SNP, and thus the number of possible structures for an LD module is equal to the number of SNPs in the module. In the exhaustive search strategy, we can search for the core SNP in linear time complexity, proportional to the number of SNPs in the module. In the forward strategy, we consider the situation of adding a SNP into an LD module, and determine the structure by comparing the likelihood values of two cases: (1) the added SNP is a peripheral SNP, and (2) the added SNP is the core SNP. This can be done in constant time complexity. In the backward strategy, we consider the situation of removing a SNP from the module. If the removed SNP is not the core SNP, we simply remove it. In the case that the deleted SNP is the core SNP, we select a new core SNP from the previous peripheral SNPs by exhaustive search, which can be done in linear time complexity, proportional to the number of SNPs remaining in the module. Since the exhaustive search strategy is straightforward and already computationally economy (linear complexity), we simply apply the exhaustive search strategy to obtain structures for LD modules.

With the module structures being obtained, we are now able to calculate the Gibbs sampler defined by Equation (8).

### Sampling the Number of Modules

We assume that the numbers of epistatic modules (*S*) and LD modules (*T*) are already known in the Gibbs sampling strategy for marker partitioning. Nevertheless the values of *S* and *T* are usually unknown in real applications. To address the uncertainty of *S* and *T*, we adopt a reversible jump Markov chain Monte Carlo (RJ-MCMC) procedure [26] as follows.

Step 1: Initialization. Assign *S* and *T* with proper positive numbers (e.g., *S* = *T* = 1).
Step 2: Gibbs sampling under the current configuration (*S*, *T*). Perform the Gibbs sampling algorithm a number of 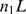 iterations using the current *S* and *T*. Record 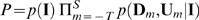.
Step 3: Propose a new configuration (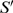, 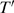). *S* and *T* are selected at random for updating. Suppose that *S* is selected, propose to increase *S* by 1 (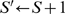) with probability 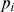 or decrease *S* by 1 (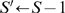) with probability 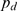, where 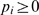, 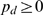, and 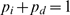. To improve the sampling efficiency, we skip the increment of *S* (or *T*) if there are empty modules and the decrement of *S* (or *T*) if it is equal to 1.
Step 4: Gibbs sampling under the proposed configuration (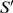, 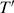). Perform the Gibbs sampling algorithm a number of 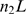 iterations using the proposed 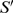 and 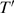. Record 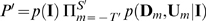.
Step 5: Choose a new configuration. Keep the original configuration (*S*, *T*) or accept the new configuration (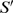, 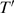) according to the acceptance probability, which is calculated as
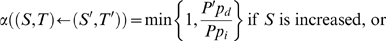


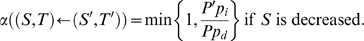

Step 6: Keep repeating steps 2 to 5 all along with the Gibbs sampling procedure for the posterior distribution of **I**.

With a sufficient number of the above RJ-MCMC sampling procedure being repeated, the Markov chains for *S* and *T* could achieve their stable distributions. In our studies, we use 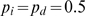, 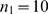, and 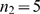.

### Statistical Significance of Epistatic Modules

The RJ-MCMC procedure samples the posterior distributions of the numbers of epistatic and LD modules, while the Gibbs sampling algorithm gives us the posterior probability that a locus belongs to a module and enables us to sample the indicators with the use of their conditional distributions in a sequential way. Starting from an initial (random) assignment of the indicators, the Gibbs sampling procedure simulates a Markov chain whose stationary distribution follows the distribution of the indicator vector. When the Markov chain reaches its stationary distribution after a number of burn-in iterations, we record candidate epistatic modules and their posterior probabilities. The posterior probability of an epistatic module represents the strength that the module is associated with the disease and thus can be directly used to make statistical inference. For example, biologists can select epistatic modules with top posterior probabilities for further functional analysis or biological experiments. Nevertheless, the statistical significance of epistatic modules might be more desired by geneticists. We therefore provide in the following parts a permutation test method and a “selection-testing-correction” approach for assessing the statistical significance of candidate epistatic modules.

#### Permutation test

For a candidate epistatic module, we need to test *H*_0_: the module is not associated with the disease versus *H*_1_: the module is associated with the disease. The posterior probability of a candidate module represents its strength of association with the disease and can serve as a test statistic for hypothesis testing. We therefore propose the following permutation test method on the basis of posterior probabilities of candidate epistatic modules.

Step 1: Apply the sampling procedure to the original case-control data with a certain parameter setting. Record candidate epistatic modules, their module sizes, and their posterior probabilities. Here, the size of a module refers to the number of SNPs included in the module.
Step 2: Permute the case-control data by shuffling the case-control labels. Apply the sampling procedure with the same parameter setting to the permuted data. Record the maximum posterior probability of sampled epistatic modules for each module size.
Step 3: Repeat the above Step 2 a number of *N* times to obtain *N* maximum posterior probabilities for each module size.
Step 4: For each candidate epistatic module (suppose its size is *s*) sampled from the original case-control data, count the number of times that the *N* maximum posterior probabilities for module size *s* are greater than or equal to the posterior probability of the candidate module and divide this count by *N* to obtain a *p*-value for the module.

#### Selection-testing-correction

Although the permutation test method can well control the type I error at the expected level, it is computationally very expensive. To alleviate the computational burden, we propose the following “selection-testing-correction” approach that uses the standard Chi-squared test with Bonferroni correction to assess the statistical significance of candidate epistatic modules.

Step 1: Selection. Apply the Gibbs sampling procedure to the original data. Collect candidate epistatic modules whose posterior probabilities are higher than a predefined threshold.
Step 2: Testing. Apply the Chi-squared test to the selected modules and obtain their *p*-values. In this procedure, the *p*-value for a module is calculated by applying the Chi-squared test to check the full interaction of core SNPs in the module.
Step 3: Correction. Apply the Bonferroni correction to the above *p*-values by multiplying them with the number of all possible tests. For a module with *c* core SNPs, this number is 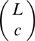, where *L* is the total number of SNPs in the original case-control data.

In the above selection-testing-correction approach, we define the statistical significance of a module as the Bonferroni corrected Chi-squared *p*-value of the full interaction of core SNPs in the module. There are several reasons to use such a definition. First, according to our genetic model for complex diseases, it is the core SNPs that contribute to the disease risk rather than the peripheral SNPs that are in LD with the core SNPs. It is therefore natural that we assess the statistical significance of a module on the basis of the core SNPs. Second, in practice, the introduction of redundancy of peripheral SNPs in the test procedure will make the *p*-values much more conservative, because in the calculation of the nominal (raw) *p*-values, the introduction of peripheral SNPs usually increases the degrees of freedom greatly but does not increase the values of the Chi-squared statistics much. Therefore the nominal *p*-values often tend to be relatively larger. Finally, in the Bonferroni correction procedure, the inclusion of peripheral SNPs makes the number of all possible tests even larger, and thus the correction for multiple testing is more severe. According to our experiments, the selection-testing-correction approach is computationally very economy and can achieve similar performance as the permutation test method.

After epistatic modules being identified by either of the above methods, one may follow the convention in association studies to claim some SNPs as representatives of epistatic modules. Intuitively, core SNPs in the identified module can well serve as such representatives, as we shall see in the simulation studies. Nevertheless, we suggest that users of our method also look at peripheral SNPs besides the attention on core SNPs, because peripheral SNPs that are in LD with core SNPs also provide useful information for the understanding of how the disease susceptibility is being affected, as we shall see in the application of our method to the real AMD data.

### Web Resources

The URL for the software presented herein is as follows: http://bioinfo.au.tsinghua.edu.cn/epiMODE

## Results

### Simulation Studies

#### Disease models

In order to test the power of the proposed approach in the identification of SNPs that are associated with disease risks, we design seven disease models with different characteristics, as illustrated in Table 1. Model 1 contains two disease loci, each of which contributes to the disease risk independently. Model 2 is similar to model 1, except that the disease risk increases only when both loci have at least one disease allele. Model 3 contains two disease loci, in which the additional disease allele at each locus does not further increase the disease risk. Model 4 contains two disease loci and assumes that the disease allele in one locus has the main effect on the disease risk. When disease alleles in both loci are present, however, the effect is inversed. Model 5 has two disease loci in which one locus has none-zero marginal effect to the disease risk while the other has absolutely no marginal effect. Model 6 is composed of four disease loci, partitioned into two epistatic modules, each of which contains two loci and has the same characteristics as model 4. Model 7 is composed of four disease loci, partitioned into two epistatic modules, each of which contains two loci and has the same characteristics as model 5.

**Table 1:** Relative risk for combinatory genotypes of disease models.

Disease Model	Locus A	Locus B		
		BB	Bb	bb
Model 1	AA	1	1+f	(1+f)^2^
	Aa	1+f	(1+f)^2^	(1+f)^3^
	aa	(1+f)^2^	(1+f)^3^	(1+f)^4^
Model 2	AA	1	1	1
	Aa	1	(1+f)^2^	(1+f)^3^
	aa	1	(1+f)^3^	(1+f)^4^
Model 3	AA	1	1	1
	Aa	1	1+f	1+f
	aa	1	1+f	1+f
Model 4	AA	1	1	1
	Aa	f	1/f	1/f
	aa	f	1/f	1/f
Model 5	AA	1/(1-MAF)^2^	0	1/(1-MAF)^2^
	Aa	0+f	1/(1- MAF^2^-(1-MAF)^2^)+f	0+f
	aa	0+f	0+f	0+f

For each of the seven disease models, we simulate eight sub-models that have different parameter settings. In the first four of them, we assume that the real causative disease loci are un-genotyped, and each of them is in linkage disequilibrium (*r* ^2^ = 0.7, see Text S1 for the calculation of *r* ^2^) with a genotyped locus (marker) that is observable in the data. In the other four, the disease markers are the real causative disease loci themselves (or in *r* ^2^ = 1 LD). The minor allele frequencies (MAF) for the disease markers (the same with the corresponding real causative disease loci) are 0.05, 0.1, 0.2, and 0.5. Note that the causative disease loci are independent. For model 1, the marginal effect size (see Text S1 for the definition) for locus A is set to 0.3, and the disease prevalence is set 0.1. For model 2, the marginal effect size for locus A is set to 0.5, and the disease prevalence is set to 0.01. For model 3, the marginal effect size for locus A is set to 1.0, and the disease prevalence is set to 0.1. For model 4 and 6, the parameter *f* for the relative risk model that adjusts the effect of disease genotypes relative to the wild ones is set to 3.0, and the disease prevalence is set 0.01. For model 5 and 7, the marginal effect size for locus A (two loci, each of which is in an epistatic module for model 7) is set to 0.5, and the disease prevalence is set to 0.005.

For each disease model, we simulate 100 data sets for a sub-model. Each data set contains 1,000 cases and 1,000 controls, in which 1,000 markers are genotyped for each subject. The random markers (markers that are not associated with the disease) are also independent. The minor allele frequency (MAF) for each random marker is chosen uniformly in [0.05, 0.5]. The detailed information for generating simulated data for a disease model with determined parameters is discussed in Text S1.

#### Comparison with existing methods

In order to illustrate the performance of the proposed method, we implement *epi*MODE, BEAM [23], stepwise logistic regression method [27], and the classical single-locus Chi-squared test. BEAM uses a Bayesian model with the Metropolis-Hastings algorithm to partition disease loci into a group that is composed of loci “contributing independently to the disease” and a group contains loci “jointly influence the disease risk” [23]. The stepwise logistic regression method is a two-state strategy. In the first stage, the most significant 10% SNPs are chosen according to their marginal effects. In the second stage, all two-way interactions of the chosen 10% SNPs are enumerated and tested for statistical significance, with marginal effects of the loci being excluded [27]. Besides these two methods, the classical single-locus Chi-squared test with two degrees of freedom is used as a benchmark. The details of these methods are given below.

For the single-locus Chi-squared test, we perform a family of Chi-squared tests (each for a single SNP) for each simulated data set. In each test, the null hypothesis is that the SNP under test is not associated with the disease, and the alternative hypothesis is that the SNP is associated with the disease. To account for the multiple testing problem, we apply the Bonferroni correction to control the family-wise error rate (FWER) at the predefined significance level (0.05). We claim that a SNP as being identified if the corrected *p*-value for the SNP is less than this significance level.

For the stepwise logistic regression method, we perform a family of logistic regressions with likelihood ratio tests (each for a pair of SNPs screened out via the single-locus scan) for each simulated data set. In each likelihood ratio test, the null hypothesis is that the interaction of the pair of SNPs under investigation is not associated with the disease, and the alternative hypothesis is that the interaction of the pair of SNPs is associated with the disease. To account for the multiple testing problem, we apply the Bonferroni correction to control the FWER at the predefined significance level (0.05). We claim both SNPs in a pair as being identified if the corrected *p*-value for the pair is less than this level. The details of this method are given in [27].

For BEAM, a Bayesian approach with a Metropolis-Hastings algorithm is first applied to each simulated data set to screen out a small number of candidate SNPs, and then all one-, two-, and three-way interactions of the candidate SNPs are checked with the use of a B-statistic [23]. In each test, the null hypothesis is that the SNP (or interaction of SNPs) under investigation is not associated with the disease, and the alternative hypothesis is that the SNP (or interaction of SNPs) is associated with the disease. To account for the multiple testing correction problem, the Bonferroni correction is also applied to control the FWER at the predefined significance level (0.05). We claim all SNPs in an interaction as being identified if the corrected *p*-value for the interaction is less than this level. The details of this method are given in [23].

For *epi*MODE, the selection-testing-correction scheme is adopted. For each simulated data set, we set all Dirichlet hyper-parameters as 0.5 and the prior probability that a SNP belongs to an epistatic or LD module as 0.01. We run 200*L* (*L* is the number of SNPs in the data set) iterations of the Gibbs sampling procedure, in which the first half is taken as burn-in, and the second half is used to record candidate modules and their posterior probabilities (in every *L* iterations). In the selection step, all candidate modules with posterior probabilities higher than a predefined cut-off (0.20 in our studies) are screened out. In the testing step, we apply a Chi-squared test to each selected module and obtain its *p*-value, as described in the method section. In the correction step, we again apply the Bonferroni correction to control the FWER at the predefined significance level (0.05). If the corrected *p*-value of a module is less than this level, we report the module as being identified and further claim core SNPs in the module as being identified.

For each of the above methods, we count a positive if all disease-associated SNPs in a simulated data set are identified, and we calculate the power of the method for a sub-model as the proportion of positives in all simulated data sets for the sub-model (100 data sets for each sub-model in our simulation studies). Therefore, powers are calculated at the same significance level (0.05) for all methods (verification is provided in Text S1). This scheme for comparing power is also used in existing literature [23],[27].

**Figure 2:**
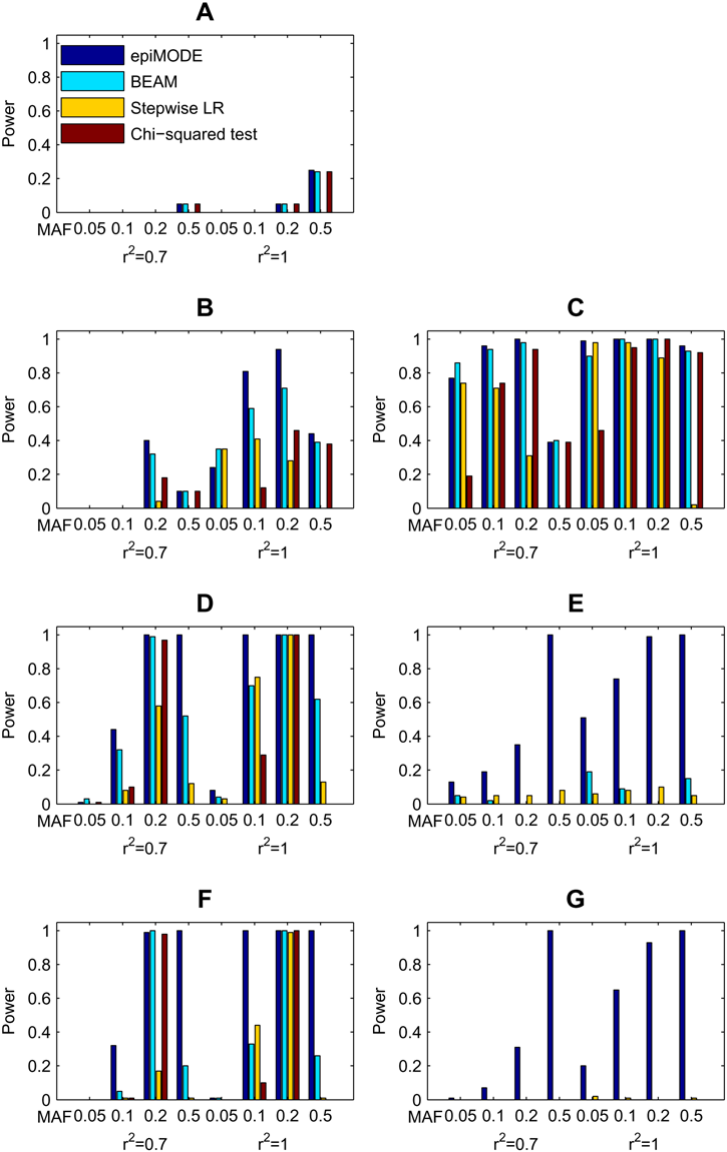
Comparison of *epi*MODE, BEAM, stepwise logistic regression (LR), and the classical single-locus Chi-squared test on seven disease models (A–G). For each parameter setting, the power is calculated as the proportion of simulated data sets in which all markers associated with the causative SNPs are indentified at the significance level 0.05 after Bonferroni correction. Each data set contains 1,000 cases and 1,000 controls, in which 1,000 markers are genotypes for each subject. The absence of bars stands for zero power.

The comparison of the powers for the four methods is shown in Figure 2. For model 1 where the disease loci have no epistatic interactions, the stepwise logistic regression method has no power at all, because the marginal effects of the loci is excluded in the second stage. The other three methods get similar powers, while *epi*MODE performs slightly better than the other two (Figure 2A). For models 2 and 3, the two disease-associated SNPs have approximate multiplicative and dominant interactions, respectively. As a result, these loci have non-negligible marginal effects and can thus be detected by all of the four methods, though the proposed *epi*MODE approach generally got moderately higher power than the others (Figures 2B and 2C). For models with moderate epistasis (models 4 and 6), *epi*MODE outperforms BEAM, and BEAM in turn outperforms the stepwise logistic regression and the single-locus Chi-squared test (Figures 2D and 2F). For models with strong epistasis in which at least one locus has no marginal effect (models 5 and 7), *epi*MODE significantly outperforms the stepwise logistic regression method. BEAM got a little power when there is only one epistatic module (model 5) but lost all the power when there are two modules in the model (model 7). The single-locus Chi-squared test has no power at all no matter what parameters are selected for the model (Figures 2E and 2G).

Although both the proposed *epi*MODE approach and the existing BEAM method rely on the Bayesian inference principle, the fact that the proposed method achieves significantly higher power than BEAM results from the following two major reasons. First, the Gibbs-sampling strategy that updates one locus at a time conditional on the current status of other markers may be more suitable for the Bayesian model used here than the Metropolis-Hastings algorithm that is used by BEAM. Because the dimension of the indicator vector that represents the property of each marker is very high, most of the search space has very low probability density. Consequently, if we randomly choose a vector that has several factors differing from the current indicator vector, the probability that the random walk will be rejected is very high, thereby resulting to an inefficient sampling process. Second, and may be more fundamental for real human complex diseases rather than that in our simulation studies where the models used are relatively simple, the proposed Bayesian partition model takes multiple epistatic and LD modules into consideration and is suitable for different disease models, while BEAM could be seen as, in some sense, a special case of our model in which the number of epistatic modules is equal to two, and the number of LD modules is equal to zero. With this understanding, if a case-control data set is sampled from a disease model that has more than two epistatic modules with more than one locus in each module, it is almost impossible for BEAM to identify all modules when the marginal effects of the loci are subtle.

In contrast to the *epi*MODE approach, the stepwise logistic regression method loses its power in both stages. In the first stage, the stepwise logistic regression does not account for epistatic interactions, while in the second stage the marginal effect is excluded from the test statistic. Results for model 1 and model 5 illustrated these disadvantages, respectively. The proposed approach, however, tends to assign loci with stronger marginal effects into epistatic modules and then attract loci that have interactions with the already assigned loci into the same module. Since both marginal and interactive effects are utilized effectively in the Gibbs sampling procedure, the proposed approach results in significantly higher power than the stepwise logistic regression in almost all simulation models.

Note that we set all Dirichlet hyper-parameters to 0.5 and use the sampling strategy to obtain module structures. A detailed analysis shows that *epi*MODE is robust to the selection of Dirichlet hyper-parameters (Text S1).

#### Performance of the permutation test method

In the above comparison studies, we use the selection-testing-correction approach to assess statistical significance of epistatic modules. Although this approach can greatly reduce the computational burden, it remains interesting to see whether this approach can achieve comparable performance as the permutation test method. We therefore compare the powers of the permutation test method and the selection-testing-correction approach.

The power for the selection-testing-correction approach is calculated using the method described in the previous section. The power for the permutation test method is calculated as follows. For each simulated data set, we run 200*L* (*L* is the number of SNPs in the data set) iterations of the Gibbs sampling procedure, in which the first half is taken as burn-in, and the second half is used to record candidate epistatic modules and their posterior probabilities (in every *L* iterations). After this, we generate 1,000 permuted data sets for each simulated data set by shuffling the disease labels. For each permuted data set, we also run the Gibbs sampling procedure (200*L* iterations) and record the maximum posterior probability for each module size. Finally, for each candidate epistatic module (suppose its size is *s*), we calculate the proportion that the recorded maximum probabilities for module size *s* are greater than or equal to the posterior probability of the candidate module to obtain its *p*-value. If the *p*-value of a module is less than a predefined significance level (0.05), we report the module as being identified and claim core SNPs in the module as being identified. If all disease-associated SNPs in a simulated data set are identified, we count a positive. The power of the permutation test method for a sub-model is then calculated as the proportion of positives in all simulated data sets for the sub-model (100 data sets for each sub-model in our simulation studies).

**Figure 3:**
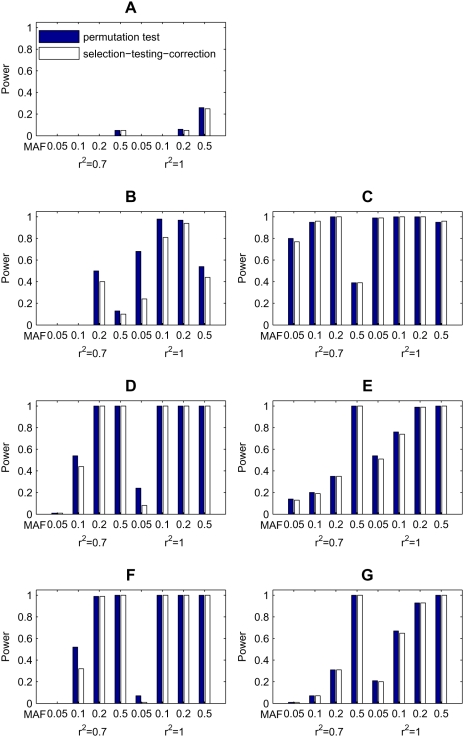
Comparison of the two methods for obtaining statistical significance for epistatic modules.

The comparison of the powers of the two methods is shown in Figure 3. We can see from the figure that the powers of the two methods are very close to each other for most situations, suggesting that both approaches work well. We also notice that the permutation test method can achieve slightly higher power than the Chi-squared test approach especially for low MAFs and/or when the disease-associated SNPs themselves are in LD (*r* ^2^ = 0.7 in the simulation studies) with the un-genotyped causative SNPs. This observation suggests that the permutation method using posterior probability may better utilize the LD information to achieve a high power, as will be analyzed in the next section.

#### Impact of peripheral SNPs

In the above simulation studies, we assume independence between genotyped SNPs. In real genome-wide association studies, however, it is common that a SNP may be in LD with some other SNPs. We use model 5 as an example to simulate this situation and to demonstrate the capability of our approach in dealing with the existence of LD between SNPs.

In the original model 5, there is an epistatic module in which two disease susceptibility SNPs contribute to the disease risk through their interactive effects. In other words, there exist two core SNPs and no peripheral SNPs in this module. To simulate the existence of peripheral SNPs, we extend this model by adding two peripheral SNPs, each in LD (*r* ^2^ = 0.7) with a core SNPs. Consequently, we have in the extended model an epistatic module that is composed of two LD sets, each containing a core SNP and a peripheral SNP. To simulate the existence of LD modules, we generate four SNPs with MAFs being 0.05, 0.1, 0.2, and 0.5, respectively. For each of these SNPs, we further add a SNP in LD (*r* ^2^ = 0.7) with it. Each added peripheral SNP has the same MAF as the corresponding core SNP. As a result, we have four LD modules in the extended model, each containing a core SNP and a peripheral SNP. Since ten more SNPs (two for the epistatic module and eight for LD modules) are added in the above modification, we delete ten disease-unassociated SNPs at random to maintain the total number of SNPs as 1,000.

**Figure 4:**
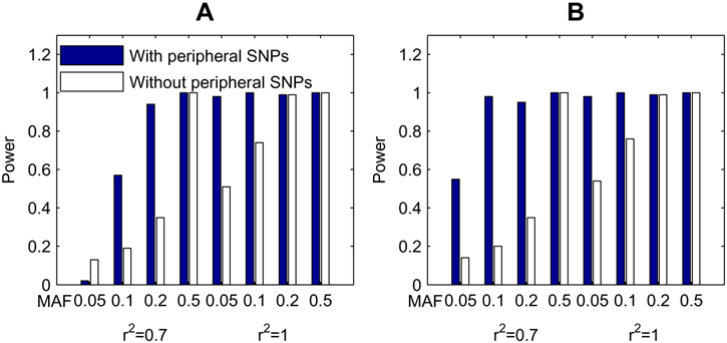
Performance of *epi*MODE on disease models with and without peripheral SNPs. (A) Results of the selection-testing-correction approach. (B) Results of the permutation test method. The original model 5 is used as the disease model without peripheral SNPs. The extended model 5 in which each of the two core SNPs has a peripheral SNPs is used as the disease model with peripheral SNPs.

The performance of *epi*MODE in the detection of the two disease susceptibility SNPs for the original and the extended models under different parameter settings is compared in Figure 4, in which Figure 4A shows powers of the selection-testing-correction approach, and Figure 4B shows powers of the permutation test method. In general, *epi*MODE achieves higher power with the addition of SNPs that are in LD with the disease susceptibility SNPs. Particularly, this observation becomes more distinct for parameter settings in which the powers of the original model are quite low (MAFs = 0.05 and 0.1). In other words, *epi*MODE can be more powerful in detecting disease susceptibility SNPs when there exist some other SNPs that are in LD with the disease susceptibility ones.

The power of *epi*MODE with the selection-testing-correction procedure depends on both the posterior probabilities of epistatic modules given by the sampling procedure and *p*-values calculated by the Chi-squared test with Bonferroni correction. The improvement in power when peripheral SNPs included is mainly due to the improvement in the posterior probabilities of the modules. It is one of the advantages of our Bayesian model that it can describe the LD between core and peripheral SNPs and then utilize this information to detect the modules. The inclusion of peripheral SNPs in a module increases the likelihood of the observed case-control data and consequently increases the posterior probability of the module in the sampling procedure, making the module easier to be screened out in the selection step. As a result, the power is improved. Nevertheless, the Chi-squared statistic, which does not account for LD, may limit the improvement in power in the testing and correction procedures. As we can see from Figure 4A, although the powers for most sub-models are improved when peripheral SNPs are included, the powers for sub-models in which the disease-associated SNPs are in LD (*r* ^2^ = 0.7) with the un-genotyped causative SNPs are in general not higher than the powers for sub-models in which the disease-associated SNPs are the causative SNPs themselves (*r* ^2^ = 1). This is natural because the signal of indirect association is usually harder to be detected [28].

In contrast to the selection-testing-correction approach, the improvement in power of the permutation test method purely depends on the increase in the posterior probabilities of epistatic modules in the sampling procedure and is not restricted by the post hoc Chi-squared test. Consequently, the permutation test method achieves higher power when peripheral SNPs are included in most cases, especially when the disease-associated SNPs (core SNPs) are in LD (*r* ^2^ = 0.7) with the un-genotyped causative SNPs (Figure 4B). We also notice that, the power (0.98) for the second sub-model (MAF = 0.1, *r* ^2^ = 0.7) that includes peripheral SNPs is much higher than the power (0.76) for the sixth sub-model (MAF = 0.1, *r* ^2^ = 1) that contains no SNPs in LD with the disease-associated SNPs. This observation suggests that, with the integration of LD information, *epi*MODE is capable of detecting indirect subtle associations in which the causative SNPs are un-genotyped. With the development of genotyping (and sequencing) technology, genome-wide case-control data become more and more dense, and more LD information will be supplied. *epi*MODE, with its capability of utilizing LD information, is especially suitable for this kind of data.

#### Distribution of the number of modules

We use model 7 as an example to demonstrate the performance of the RJ-MCMC procedure in addressing the uncertainty of the numbers of epistatic and LD modules. In the original model 7, there exist two epistatic modules, each containing two disease susceptibility SNPs. We extend the seventh parameter setting for model 7, in which the MAFs for all disease loci are 0.2, and the disease markers are the real disease causative loci themselves (*r* ^2^ = 1). To simulate the existence of LD modules, we generate four SNPs with MAFs being 0.05, 0.1, 0.2, and 0.5, respectively. For each of these SNPs, we further add a SNP in LD (*r* ^2^ = 0.7) with it. Each added peripheral SNP has the same MAF as the corresponding core SNP. Consequently, we have four LD modules in the extended model, each containing a core SNP and a peripheral SNP. Since eight SNPs are added in the above procedure, we delete the same number of disease-unassociated SNPs at random to maintain the total number of SNPs as 1,000.

**Figure 5:**
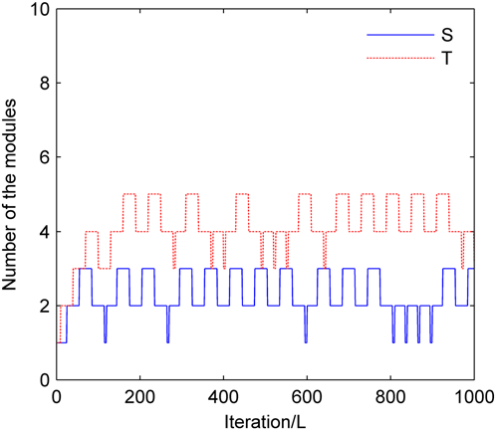
Distributions of the numbers of epistatic and LD modules. Model 7 with parameters MAF = 0.2 and *r* ^2^ = 1 is extended to include two epistatic modules and four LD modules. Results are obtained with parameters *n*_1_ = 10 and *n*_2_ = 5.

For the extended model in which *S* = 2 and *T* = 4, we apply *epi*MODE to detect the disease susceptibility SNPs, and we record the traces of the numbers of epistatic and LD modules sampled by the RJ-MCMC strategy. The results are shown in Figure 5. Starting from initial values (*S* = *T* = 1), the Markov chain gradually reaches its stationary distribution. Specifically, after the burn-in (100*L* iteration), the probabilities for *S* to be 1, 2 and 3 are 0.0389, 0.5767 and 0.3844, respectively, and the probabilities for *T* to be 3, 4 and 5 are 0.0778, 0.5889 and 0.3333, respectively. These results suggest that the RJ-MCMC strategy is effective in handling the uncertainty of the numbers of epistatic and LD modules.

### A Genome-Wide Association Study on AMD

In order to verify the capability of the proposed approach in the detection of epistatic interactions in real genome-wide association studies, we apply *epi*MODE to an Age-related Macular Degeneration (AMD) data set [20], which contains 103,611 SNPs genotyped with 96 cases and 50 controls.

**Figure 6:**
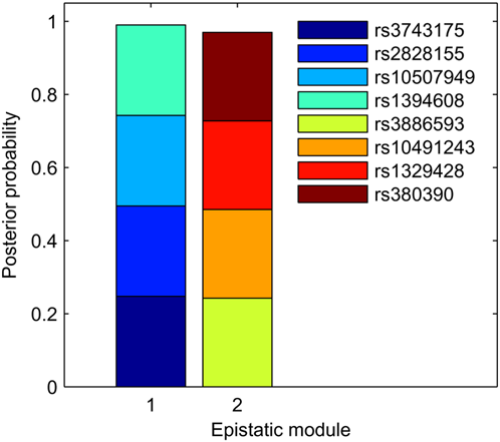
Posterior probabilities of epistatic modules identified in the AMD data set. The prior probability that a marker is involved in a module is set to 10/103611. The cutoff posterior probability for reporting an epistatic module is 0.9.

**Figure 7:**
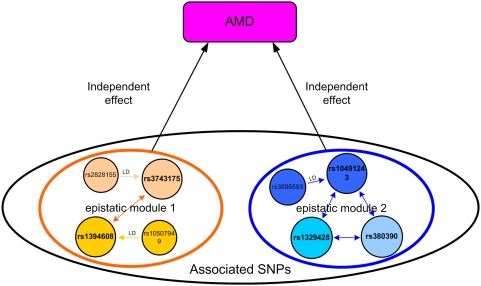
Structures of epistatic modules identified in the AMD data set. Double arrowheads connect core SNPs in epistatic modules. Single arrowheads link peripheral SNPs to core SNPs.

The authors of the original paper reported that two SNPs, rs380390 and rs1329428, were believed to be significantly associated with AMD. Our method successfully indentifies both of the two SNPs through the identification of an epistatic module that included these two SNPs (two more SNPs are also indentified in the same epistatic module, and the posterior probability of the module is above 0.9, see Figures 6 and 7). The nominal *p*-values for rs380390 and rs1329428 are 1.75×10^−6^ and 3.64×10^−6^, respectively, according to the Chi-squared test with two degrees of freedom. Our method also indentifies two novel SNPs, rs1394608 and rs3743175, by detecting an epistatic module that includes both loci (two more SNPs in LD with them are also indentified in the same epistatic module, and the posterior probability of the module is greater than 0.9, see Figures 6 and 7). The nominal *p*-values for these two SNPs are 8.81×10^−5^ and 1.76×10^−3^, respectively, according to the Chi-squared test with two degrees of freedom. Note that the *p*-value for the combination of rs1394608 and rs3743175 is 7.39×10^−7^, while the *p*-value for the combination of rs380390 and rs1329428 is only 1.84×10^−5^, according to the Chi-squared test with eight degrees of freedom.

**Figure 8:**
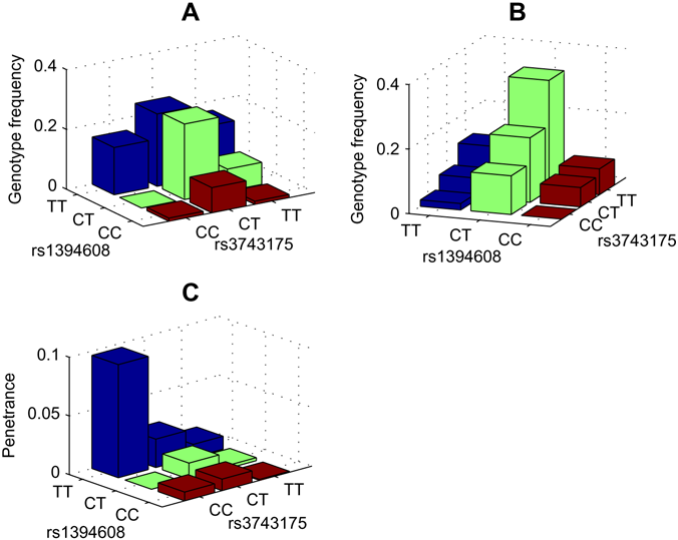
Distributions of genotypes of rs1394608 and rs3743175 in the AMD data set and the penetrance estimated for combinatory genotypes of these two SNPs. (A) The distribution of genotypes of rs1394608 and rs3743175 in the case population. (B) The distribution of genotypes of rs1394608 and rs3743175 in the control population. (C) The estimated penetrance for the combination of rs1394608 and rs3743175, assuming that the disease prevalence is 0.01. The relative values for the penetrance maintain the same when the population prevalence is alternatively assumed. It is shown that rs1394608 could be seen as the dominant-effect locus for the disease risk, and its effect is strongly regulated by rs3743175.

The distributions of the combination of rs1394608 and rs3743175 in cases and controls are shown in Figures 8A and 8B, respectively. According to Chi-squared tests with four degrees of freedom, these two SNPs are independent in controls (*p*-value = 7.50×10^−1^) and dependent in cases (*p*-value = 3.27×10^−3^). We also infer the genotype frequencies of the combination of these two SNPs according to their distributions in controls and the Hardy-Weinberg equilibrium (HWE), and we further infer the penetrance for the combination of these two SNPs according to their distributions in cases and the inferred genotype frequencies, as shown in Figure 8C (see Text S1 for details of inferring genotype frequencies and the penetrance). The penetrance of genotypes of rs1394608 differs stronger from that of rs3743175, suggesting that rs1394608 may be the dominant locus for disease susceptibility. Specifically, the homozygote TT of rs1394608 is responsible for disease risk significantly higher than the heterozygous and the other homozygous genotype. However, the effect of rs1394608 is strongly regulated by rs3743175, especially for the homozygous genotype TT of rs1394608. The penetrance for the combination genotype TT/CC (for rs1394608 and rs3743175, respectively), is 9.64×10^−2^, significantly larger than the penetrance for the combination genotype TT/CT and the penetrance for the combination genotype TT/TT. Odds ratio values in table 2 also give similar results. From the above analysis, we infer that the relationship between the combination of SNPs rs1394608 and rs3743175 and the disease risk is a classic epistatic interaction, in which one dominant variant locus (rs1394608) is regulated by the other locus (rs3743175). In the following part, we perform functional analysis of these two SNPs.

**Table 2:** Odds ratios for rs3743175 in *SCAPER* and rs1394608 in *SGCD*.

SNP		*SGCD* rs1394608		
		TT	TC	CC
*SCAPER* rs3743175	TT	10.67 (0.98, 115.68)	1.65 (0.16, 17.47)	1.00
	TC	30.67 (2.52, 373.55)	9.60 (0.95, 96.92)	10.67 (0.82, 138.22)
	CC	60.00 (3.04, 1185.03)	0.00	Inf

Odds ratios with 95% confidence intervals in parentheses were calculated to compare each genotypic combination to the baseline of homozygosity for common allele at rs3743175 and less common allele at rs1394608 (TT/CC).

AMD is the primary cause of irreversible visual loss in the Western world [29]. The clinical hallmark of AMD is pathological extracellular deposits in retinal called drusen. Previous single-locus studies have identified the complement factor H (*CFH*) and the HtrA serine peptidase 1 (*HTRA1*) as two major risk genes for AMD [30]–[32]. Despite the complex etiology of AMD, no significant epistasis has been identified by BEAM in the genome-wide case-control data used in this study.

The most significant epistatic effect we identified is between SNPs rs1394608 and rs3743175. Interestingly, there is another SNP rs2828155 with exactly the same genotype distribution as rs3743175 among all case/control samples (rs2828155 is also detected by *epi*MODE in the same module with rs1394608 and rs3743175, see Figures 6 and 7); therefore the epistasis may also exist between rs1394608 and rs2828155.

rs1394608 resides within the intron of *SGCD*, a gene located on chromosome 5q33-34, which has been implicated in AMD [33],[34] and predispose to drusen formation [35]. *SGCD* is the delta subunit of the sarcoglycan complex, a component of the dystrophin-glycoprotein complex, linking the cytoskeleton to the extracellular matrix. The sarcoglycan complex involves in plasma membrane deposition, and the co-expression of *SGCD* and *SGCB* (beta subunit) is responsible for delivery to and retention of sarcoglycan complex at the cell surface [36]. Defects in *SGCD* are the cause of limb-girdle muscular dystrophy type 2F (*LGMD2F*) and dilated cardiomyopathy 1L (*CMD1L*). The detected SNP rs1394608, together with all 16 SNPs of strong LD (r^2^>0.8) within 1 Mb neighboring region, are all significantly associated with the expression of *FBLN1* (*p*-value<1×10^−7^, according to [37]), a gene belongs to the fibulin family of extracellular matrix proteins. Other members (*FBLN3*, *FBLN5*, *FBLN6*) of the family have been associated with AMD [38]–[41], and various evidences support that *FBLN1* may also play a role in AMD [39], [42]–[45]. Specifically, *FBLN1* can act as a cofactor for the matrix metalloprotease *ADAMTS1* and play important roles in the degradation of proteoglycans by *ADAMTS1* during pathological conditions induced by inflammatory processes [46]. Therefore variants in *SGCD* may lead to AMD in a similar way to *HTRA1*, which may regulate the degradation of extracellular matrix by facilitating access of other degradative matrix enzymes, such as matrix metalloproteinases to their substrates [47].

rs3743175 resides within the intron of *SCAPER*/*ZNF291*, a gene located on chromosome 15q24. Iyengar et al [34] have also identified linkage signal for a marker in the nearby locus 15q21. A weak linkage signal on chromosome 15q has also been observed in another full-genome scan [48]. Further, translocation of 15q24 had been found in a patient with visible disc drusen [49]. Sequence analysis [50] identified in *SCAPER* an unstable non-coding tandem repeat, an important form of mutation responsible for several neurological, neurodegenerative and neumuscular disorders [51]. The detected SNP rs3743175 has the strongest association with the expression of the gene itself (*p*-value = 9.5×10^−14^).

The mechanism for the epistasis between rs1394608 in *SGCD* and rs3743175 in *SCAPER* is unclear. We speculate that *SCAPER* may exert influence to *SGCD* susceptibility through the regulation of aging process, as evidences show both genes are involved in cell cycle regulation and DNA repair. eQTL analysis [37] shows that rs1394608 is significantly associated with the expression of *RAD9B* (*p*-value<1×10^−7^), a novel component of the 9-1-1 cell-cycle checkpoint response complex [52], while rs3743175 is significantly associated with the expression of *SCAPER*, a novel regulator of cell cycle progression [53]. We also find that the DNA repair gene *ERCC6*, which plays roles in the aging process and predisposes to AMD [54], is significantly (*p*-value<1×10^−4^) co-expressed with *SGCD* and *SCAPER* across more than 40 human tissues [55]. Co-expression analysis also finds that both *SGCD* and *SCAPER* are significantly correlated with *MASP1* (*p*-value<1×10^−2^) and *MASP2* (*p*-value<1×10^−5^), activators of the complement pathway. Together with the report of synergic effect between *ERCC6* and *CFH* in predisposing AMD [54], the above analyses suggest clues for the link between the aging component and the immune component in the etiology of AMD.

The third SNP rs2828155 locates in an about 4 Mb intergenic region in chromosome 21q21.1, a region has also been implicated in AMD [35]. *ADAMTS1* and *ADAMTS5* lie about 4 Mb downstream of the SNP. There is possibility that rs2828155 may regulate the expression of these two enzymes, and then the epistasis between rs2828155 and rs1394608 is more straightforward: rs2828155 regulates the enzyme *ADAMTS1* and rs1394608 regulates *FBLN1*. As *FBLN1* can act as a cofactor of *ADAMTS1* and plays an important role in the degradation of proteoglycans by *ADAMTS1* during pathological conditions induced by inflammatory processes [46], it is possible that rs2828155 and rs1394608 have epistatic effect in AMD. Linkage signals for AMD from the two loci have been detected in the same linkage scan [35].

In summary, our association study suggests the existence of epistasis in AMD, while the functional analysis provides new insights for the understanding of the epistasis from the biological point of view. Certainly, further work, especially experimental verification of the above epistasis, is necessary in order to confirm the roles of the identified SNPs and their epistasis in AMD.

### A Genome-Wide Association Study on Parkinson's Disease

We further apply our approach to a genome-wide case-control data set of Parkinson's disease [21],[22], which contains 408,803 SNPs genotyped with 270 cases and 271 controls. With the use of *epi*MODE, we identify 12 independent contributing markers with posterior probabilities of associations greater than or equal to 0.9. The *p*-values for these markers, obtained by Chi-squared tests with two degrees of freedom, are shown in Table 3, which suggest that 7 out of the 12 markers are statistically significantly. The original paper [21] only tests SNPs that give successful genotypes in more than 95% samples. As a result, the significant markers identified by our method are all excluded. In our analysis, we run our method on the original data without discarding any SNP.

**Table 3:** *p*-values for SNPs with posterior probabilities greater than or equal to 0.9.

Index in the data	dbSNP ID	*p*-value
6321	rs12069733	1.77×10^−7^
52635	rs6757197	6.89×10^−7^
122959	rs1504212	4.31×10^−8^
142148	rs557074	4.60×10^−8^
172163	rs850084	2.20×10^−8^
177104	rs6460033	4.45×10^−7^
201738	rs7846412	5.13×10^−7^
215060	rs10963676	5.46×10^−8^
234666	rs2666781	5.16×10^−7^
240134	rs4746675	8.03×10^−8^
257981	rs12364577	9.21×10^−8^
358054	rs9952724	7.78×10^−10^

The nominal *p*-value for Bonferroni corrected significant level of 0.05 is 1.22×10^−7^.

The fact that no interaction effect is detected may be partly due to the disease model itself, which may have no strong interaction effects. Another reason may be the missing genotype problem that aggravates the insufficiency of the sample size in mapping epistatic effects. In the detection of a *k*-locus interaction, if the genotype missing rate is 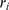 (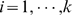) for each locus, the expected percentage of samples that could be used is only 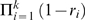, which decreases fast with *k*, the number of loci in the interaction, and makes the power for detecting high-order interactions even lower.

## Discussion

In this paper, we explicitly define epistatic modules as basic genetic units that influence the disease susceptibility and put forward a Bayesian marker partition model to explain the observed case-control data. We develop a Gibbs sampling strategy to simulate the posterior distributions that markers belong to epistatic modules and further resort to hypothesis testing to screen out statistically significant modules. We extensively assess the effectiveness of the proposed *epi*MODE approach. In simulation studies, *epi*MODE significantly outperforms all other methods. In the application to the Age-related Macular Degeneration (AMD) data, *epi*MODE successfully identifies two loci that are known to be associated with the disease, and suggests the epistatic interaction of two other loci. In the application to the Parkinson's disease data, *epi*MODE identifies seven loci that might contribute to the disease susceptibility.

The success of the proposed approach can be attributed to a combination of several aspects. First, with the explicit definition of epistatic modules, our approach is able to capture patterns of epistatic interactive effects of multiple loci in a native way. Second, the incorporation of LD information between markers in epistatic modules greatly improves the power of our method in the detection of indirect subtle associations. Third, the introduction of LD modules further minimizes the possibility of assigning disease-unassociated loci into epistatic modules. Fourth, the Gibbs sampling strategy is effective in obtaining the posterior distributions that disease-associated loci belong to epistatic modules. Finally, the native identification of interactive effects of multiple loci (epistatic modules) instead of enumerating combinations of SNPs makes our approach capable of and suitable for dealing with large scale case-control data.

Our marker partition model is proposed from the Bayesian perspective, and thus a natural advantage of this model is its capability of incorporating prior biological knowledge about individual SNP markers, such as their locations (e.g., coding region, promoter region, etc.), genotype frequencies, and LD information. Nevertheless, some parts in our approach are not formulated from the pure Bayesian perspective, mainly for the consideration of reducing the computational burden. Structures of epistatic modules are represented by results of searching procedures (exhaustive/sampling/greedy) rather than averaged over all possible structures according to their posterior probabilities because this part is heavily used by the up-level Gibbs sampling algorithm. The standard Chi-squared test with Bonferroni correction is also much more computationally economy than the permutation test method. Such efforts for greatly reducing the computational burden are necessary in handling large scale case-control data sets, which may contain more than 500,000 SNPs and have been very common in recent genome-wide association studies.

Certainly, the proposed approach can further be improved from the following directions. First, the assumption of equal values for all Dirichlet priors in the Bayesian marker partition model is obviously for the purpose of seeking for simplicity. Although different priors do not yield very different results, careful selection of priors is still worth investigating. One possibility is to systematically minimize the impact of priors using techniques such as prior annealing [56]. Another possibility is to select priors that reflect existing biological knowledge, such as the rich genotype frequency and LD information from the International HapMap Project [57],[58]. Second, although our experience suggests that the current Gibbs sampling strategy works well, some sophisticated sampling strategy such as the “split-merge” algorithm [59] might be incorporated to further improve the efficiency of the sampling strategy. Finally, currently we do not formulate the marker partitioning procedure from the viewpoint of mixture models, such as the Dirichlet process (DP) mixture model. Since a DP mixture model assumes an infinite number of mixture components and thus provides more flexibility in controlling the complexity of the model [60],[61], it would be interesting to explore the possibility of incorporating the DP mixture model into genome-wide association studies.

## Supporting Information

Text S1 Epistatic module detection for case-control studies: a Bayesian model with a Gibbs sampling strategy.
(0.21 MB PDF)
