# Supplementary material for: Epistatic Module Detection for Case-Control Studies: A Bayesian Model with a Gibbs Sampling Strategy
Source: PLoS Genet. 2009 May 1;5(5):e1000464. doi: 10.1371/journal.pgen.1000464 (PMC2669883; doi:10.1371/journal.pgen.1000464)
Supplement: Text S1 — Epistatic module detection for case-control studies: a Bayesian model with a Gibbs sampling strategy. (0.21 MB PDF) [file pgen.1000464.s001.pdf]

Epistatic module detection for case-control studies: A Bayesian model with a Gibbs sampling strategy — Supporting Information

Wanwan Tang, Xuebing Wu, Rui Jiang [*], and Yanda Li [*]

MOE Key Laboratory of Bioinformatics, Bioinformatics Division, TNLIST/Dept of Automation
Tsinghua University, Beijing 100084, China

Properties of epistatic modules

Property 1: *Epistatic modules have no overlaps between each other. Mathematically, let* $\mathbf{S}=\{\ell_1,\cdots,\ell_s\}$ *be the set of s loci that potentially contribute to the disease susceptibility. For any two epistatic modules* $\mathbf{S}_1\subseteq\mathbf{S}$, $\mathbf{S}_2\subseteq\mathbf{S}$, $\mathbf{S}_1\neq\mathbf{S}_2$, *we have* $\mathbf{S}_1\bigcap\mathbf{S}_2=\emptyset$.

Proof: Define $\mathbf{S}_{1\cap 2}=\mathbf{S}_1\bigcap\mathbf{S}_2$. We prove that $\mathbf{S}_{1\cap 2}=\emptyset$.

If $\mathbf{S}_{1\cap 2}\neq\emptyset$, we would have either $\mathbf{S}_2\subseteq\mathbf{S}_1$ or $\mathbf{S}_2\not\subseteq\mathbf{S}_1$. If $\mathbf{S}_2\subseteq\mathbf{S}_1$,

$$\begin{aligned}p(D\,|\,G_{\mathbf{S}_1}) &= \sum_{G_{\mathbf{S}_{-1}}} p(D\,|\,G_{\mathbf{S}_1},G_{\mathbf{S}_{-1}})\,p(G_{\mathbf{S}_{-1}})\\ &= \sum_{G_{\mathbf{S}_{-1}}} f_2(G_{\mathbf{S}_2})\,f_{-2}(G_{\mathbf{S}_{-2}})\,p(G_{\mathbf{S}_{-1}})\\ &= f_2(G_{\mathbf{S}_2})\sum_{G_{S_{-1}}} f_{-2}(G_{\mathbf{S}_{-2}})\,p(G_{\mathbf{S}_{-1}})\\ &= f_2(G_{\mathbf{S}_2})\,f_{1-2}(G_{\mathbf{S}_{1-2}})\end{aligned}$$

where $G_{\cdot}$ is a genotype of the corresponding locus combination, $\mathbf{S}_{-1}$ the complementary set of $\mathbf{S}_1$, $\mathbf{S}_{i_1-i_2}$ the set of loci that belong to $\mathbf{S}_{i_1}$ but not $\mathbf{S}_{i_2}$, and $f_i(\cdot)$ a function of the genotypes of $\mathbf{S}_i$. Now we have that $f_{1-2}(G_{\mathbf{S}_{1-2}})=\sum_{G_{\mathbf{S}_{-1}}} f_{-2}(G_{\mathbf{S}_{-2}})\,p(G_{\mathbf{S}_{-1}})$ is a function of the genotype of the loci that belongs to $\mathbf{S}_1$ but not $\mathbf{S}_2$. The penetrance of $\mathbf{S}_1$ is the product of a function of the genotype of a sub set of $\mathbf{S}_1$ ($\mathbf{S}_2$) and a function of the genotype of the complement of this sub set ($\mathbf{S}_{1-2}$). This is inconsistent with the assumption that $\mathbf{S}_1$ is an epistatic module.

On the other hand, if $\mathbf{S}_2\not\subseteq\mathbf{S}_1$

$$\begin{aligned}p(D\,|\,G_{\mathbf{S}_1}) &= \sum_{G_{\mathbf{S}_{-1}}} p(D\,|\,G_{\mathbf{S}_2},G_{\mathbf{S}_{-2}})\,p(G_{\mathbf{S}_{-1}})\\ &= \sum_{G_{\mathbf{S}_{-1}}} f_2(G_{\mathbf{S}_2})\,f_{-2}(G_{\mathbf{S}_{-2}})\,p(G_{\mathbf{S}_{-1}})\\ &= \sum_{G_{\mathbf{S}_{-1-(2-1\cap 2)}}} f_{-2}(G_{\mathbf{S}_{-2}})\,p(G_{\mathbf{S}_{-1-(2-1\cap 2)}})\sum_{G_{\mathbf{S}_{2-1\cap 2}}} f_2(G_{\mathbf{S}_2})\,p(G_{\mathbf{S}_{2-1\cap 2}})\\ &= f_{1-1\cap 2}(G_{\mathbf{S}_{1-1\cap 2}})\,f_{1\cap 2}(G_{\mathbf{S}_{1\cap 2}})\end{aligned}$$

[*] Corresponding authors.

Now we have $f_{1-1\cap 2}(G_{\mathbf{S}_{1-1\cap 2}})=\sum_{G_{\mathbf{S}_{-1-(2-1\cap 2)}}} f_{-2}(G_{\mathbf{S}_{-2}})p(G_{\mathbf{S}_{-1-(2-1\cap 2)}})$ is a function of the genotype of the loci belonging to $\mathbf{S}_1$ but not $\mathbf{S}_2$, and $f_{1\cap 2}(G_{\mathbf{S}_{1\cap 2}})=\sum_{G_{\mathbf{S}_{2-1\cap 2}}} f_2(G_{\mathbf{S}_2})p(G_{\mathbf{S}_{2-1\cap 2}})$ is a function of the genotype of the loci belonging to both $\mathbf{S}_1$ and $\mathbf{S}_2$. The penetrance of $\mathbf{S}_1$ is the product of a function of the genotype of a sub set of $\mathbf{S}_1$ ($\mathbf{S}_{1\cap 2}$) and a function of the genotype of the complement of this sub set ($\mathbf{S}_{1-1\cap 2}$). This is inconsistent with the assumption that $\mathbf{S}_1$ is an epistatic module.

Because $\mathbf{S}_{1\cap 2}\neq\emptyset$ is inconsistent with the truth that either $\mathbf{S}_1$ is an epistatic module, we have that $\mathbf{S}_{1\cap 2}=\emptyset$.

Property 2: *Epistatic modules are independent in both case and control populations, given that the disease susceptibility loci are in linkage equilibrium.*

Proof: Suppose that the set of disease susceptibility loci $\mathbf{S}=\{\ell_1,\cdots,\ell_s\}$ consists of m epistatic modules, that is, $\mathbf{S}=\mathbf{S}_1\bigcup\mathbf{S}_2\bigcup\cdots\bigcup\mathbf{S}_m$, $1\le m\le s$. Define $\mathbf{S}_{-i}$ as the complementary set of $\mathbf{S}_i$, $i=1,\cdots,m$, we have

$$
\begin{aligned}
p(G_{\mathbf{S}_i},G_{\mathbf{S}_{-i}}\mid D)&=\frac{p(D\mid G_{\mathbf{S}_i},G_{\mathbf{S}_{-i}})p(G_{\mathbf{S}_i},G_{\mathbf{S}_{-i}})}{p(D)}\\
&=\frac{p(D\mid G_{\mathbf{S}_i},G_{\mathbf{S}_{-i}})p(G_{\mathbf{S}_i},G_{\mathbf{S}_{-i}})p(D)}{p(D)^2}\\
&=\frac{p(D\mid G_{\mathbf{S}_i},G_{\mathbf{S}_{-i}})p(G_{\mathbf{S}_i},G_{\mathbf{S}_{-i}})\sum_{G_{\mathbf{S}_i}}\sum_{G_{\mathbf{S}_{-i}}} p(D,G_{\mathbf{S}_i},G_{\mathbf{S}_{-i}})}{p(D)^2}\\
&=\frac{p(D\mid G_{\mathbf{S}_i},G_{\mathbf{S}_{-i}})p(G_{\mathbf{S}_i})p(G_{\mathbf{S}_{-i}})\sum_{G_{\mathbf{S}_i}}\sum_{G_{\mathbf{S}_{-i}}} p(D\mid G_{\mathbf{S}_i},G_{\mathbf{S}_{-i}})p(G_{\mathbf{S}_i})p(G_{\mathbf{S}_{-i}})}{p(D)^2}\\
&=\frac{f_i(G_{\mathbf{S}_i})f_{-i}(G_{\mathbf{S}_{-i}})p(G_{\mathbf{S}_i})p(G_{\mathbf{S}_{-i}})\sum_{G_{\mathbf{S}_i}}\sum_{G_{\mathbf{S}_{-i}}} f_i(G_{\mathbf{S}_i})f_{-i}(G_{\mathbf{S}_{-i}})p(G_{\mathbf{S}_i})p(G_{\mathbf{S}_{-i}})}{p(D)^2}\\
&=\frac{f_i(G_{\mathbf{S}_i})f_{-i}(G_{\mathbf{S}_{-i}})p(G_{\mathbf{S}_i})p(G_{\mathbf{S}_{-i}})\sum_{G_{\mathbf{S}_i}} f_i(G_{\mathbf{S}_i})p(G_{\mathbf{S}_i})\sum_{G_{\mathbf{S}_{-i}}} f_{-i}(G_{\mathbf{S}_{-i}})p(G_{\mathbf{S}_{-i}})}{p(D)^2}\\
&=\frac{\sum_{G_{\mathbf{S}_i}} f_i(G_{\mathbf{S}_i})f_{-i}(G_{\mathbf{S}_{-i}})p(G_{\mathbf{S}_i})p(G_{\mathbf{S}_{-i}})\sum_{G_{\mathbf{S}_{-i}}} f_i(G_{\mathbf{S}_i})f_{-i}(G_{\mathbf{S}_{-i}})p(G_{\mathbf{S}_i})p(G_{\mathbf{S}_{-i}})}{p(D)^2}\\
&=\frac{\sum_{G_{\mathbf{S}_i}} p(D\mid G_{\mathbf{S}_i},G_{\mathbf{S}_{-i}})p(G_{\mathbf{S}_i},G_{\mathbf{S}_{-i}})\sum_{G_{\mathbf{S}_{-i}}} p(D\mid G_{\mathbf{S}_i},G_{\mathbf{S}_{-i}})p(G_{\mathbf{S}_i},G_{\mathbf{S}_{-i}})}{p(D)^2}\\
&=\frac{p(D,G_{\mathbf{S}_{-i}})p(D,G_{\mathbf{S}_i})}{p(D)^2}\\
&=p(G_{\mathbf{S}_{-i}}\mid D)p(G_{\mathbf{S}_i}\mid D)
\end{aligned}
$$

Therefore, $p(G_{\mathrm{S}} \mid D) = \prod_{i=1}^{m} p(G_{\mathrm{S}_i} \mid D)$, and consequently the epistatic modules are independent in the case population.

In control population,

$$p(G_{\mathrm{S}_i}, G_{\mathrm{S}_{-i}} \mid \bar{D}) = \frac{p(\bar{D} \mid G_{\mathrm{S}_i}, G_{\mathrm{S}_{-i}})\, p(G_{\mathrm{S}_i}, G_{\mathrm{S}_{-i}})}{p(\bar{D})}$$
$$= \frac{p(\bar{D} \mid G_{\mathrm{S}_i}, G_{\mathrm{S}_{-i}})\, p(G_{\mathrm{S}_i})\, p(G_{\mathrm{S}_{-i}})}{p(\bar{D})}$$
$$= \frac{p(\bar{D} \mid G_{\mathrm{S}_i}, G_{\mathrm{S}_{-i}})\, p(\bar{D})\, p(G_{\mathrm{S}_i})\, p(G_{\mathrm{S}_{-i}})}{p(\bar{D})^2}$$

and

$$p(\bar{D} \mid G_{\mathrm{S}_i}, G_{\mathrm{S}_{-i}})\, p(\bar{D}) - p(\bar{D} \mid G_{\mathrm{S}_i})\, p(\bar{D} \mid G_{\mathrm{S}_{-i}})$$
$$= \left(1 - f_i(G_{\mathrm{S}_i}) f_{-i}(G_{\mathrm{S}_{-i}})\right)\left(1 - \sum_{G_{\mathrm{S}_i}} \sum_{G_{\mathrm{S}_{-i}}} f_i(G_{\mathrm{S}_i}) f_{-i}(G_{\mathrm{S}_{-i}})\, p(G_{\mathrm{S}_i})\, p(G_{\mathrm{S}_{-i}})\right) -$$
$$\left(1 - \sum_{G_{\mathrm{S}_{-i}}} f_i(G_{\mathrm{S}_i}) f_{-i}(G_{\mathrm{S}_{-i}})\, p(G_{\mathrm{S}_{-i}})\right)\left(1 - \sum_{G_{\mathrm{S}_i}} f_i(G_{\mathrm{S}_i}) f_{-i}(G_{\mathrm{S}_{-i}})\, p(G_{\mathrm{S}_i})\right)$$
$$= f_i(G_{\mathrm{S}_i})\left(\sum_{G_{\mathrm{S}_{-i}}} f_{-i}(G_{\mathrm{S}_{-i}})\, p(G_{\mathrm{S}_{-i}}) - f_{-i}(G_{\mathrm{S}_{-i}})\right) +$$
$$\left(f_{-i}(G_{\mathrm{S}_{-i}}) - \sum_{G_{\mathrm{S}_{-i}}} f_{-i}(G_{\mathrm{S}_{-i}})\, p(G_{\mathrm{S}_{-i}})\right) \sum_{G_{\mathrm{S}_i}} f_i(G_{\mathrm{S}_i})\, p(G_{\mathrm{S}_i})$$
$$= \left(f_i(G_{\mathrm{S}_i}) - \sum_{G_{\mathrm{S}_i}} f_i(G_{\mathrm{S}_i})\, p(G_{\mathrm{S}_i})\right)\left(\sum_{G_{\mathrm{S}_{-i}}} f_{-i}(G_{\mathrm{S}_{-i}})\, p(G_{\mathrm{S}_{-i}}) - f_{-i}(G_{\mathrm{S}_{-i}})\right)$$

For complex disease, the disease prevalence is usually very small, we have

$$\frac{\left(f_i(G_{\mathrm{S}_i}) - \sum_{G_{\mathrm{S}_i}} f_i(G_{\mathrm{S}_i})\, p(G_{\mathrm{S}_i})\right)\left(\sum_{G_{\mathrm{S}_{-i}}} f_{-i}(G_{\mathrm{S}_{-i}})\, p(G_{\mathrm{S}_{-i}}) - f_{-i}(G_{\mathrm{S}_{-i}})\right)}{p(\bar{D} \mid G_{\mathrm{S}_i})\, p(\bar{D} \mid G_{\mathrm{S}_{-i}})} \approx 0 .$$

Thus

$$\frac{p(\bar{D} \mid G_{\mathrm{S}_i}, G_{\mathrm{S}_{-i}})\, p(\bar{D})}{p(\bar{D} \mid G_{\mathrm{S}_i})\, p(\bar{D} \mid G_{\mathrm{S}_{-i}})} \approx 1 .$$

Now we have

$$p(G_{\mathrm{S}_i}, G_{\mathrm{S}_{-i}} \mid \bar{D}) \approx \frac{p(\bar{D} \mid G_{\mathrm{S}_i})\, p(\bar{D} \mid G_{\mathrm{S}_{-i}})\, p(G_{\mathrm{S}_i})\, p(G_{\mathrm{S}_{-i}})}{p(\bar{D})^2}$$
$$= \frac{p(\bar{D}, G_{\mathrm{S}_i})\, p(\bar{D}, G_{\mathrm{S}_{-i}})}{p(\bar{D})^2}$$
$$= p(G_{\mathrm{S}_i} \mid \bar{D})\, p(G_{\mathrm{S}_{-i}} \mid \bar{D},)$$

Determination of the penetrance of the combinatory genotypes in simulation studies

For each model, there is a relative risk table in which only one parameter *f* is to be determined (see table 1 in the main text for details). Considering that real penetrance (the risk to be affected) is the product of the relative risk and another parameter that represents the value that "1" in the relative risk table stands for, there are only two parameters to be determined. In order to control the "marginal effect size" λ of the first disease locus (locus A in table 1) and the population prevalence $p(D)$, there will be two equations to solve. The definition of λ is the same as the one used in (Zhang and Liu 2007). We use *b* as the parameter that is equal to the value that "1" in the relative risk table stands for, **R** as the relative risk table, and $\mathbf{P}(i, j)$ as the genotype frequency of the locus combination with the first locus being the *i*-th genotype and the second the *j*-th.

Assuming the Hardy-Weinberg equilibrium, the genotype frequencies for each locus (locus *k*) could be determined as $f_k = \left((1-MAF_k)^2, 2MAF_k(1-MAF_k), MAF_k{}^2\right)$, given the minor allele frequency MAF_k for the locus *k*. Assuming linkage equilibrium (independent distribution) between the disease susceptibility loci, the frequency of the combinatory genotype $\mathbf{P}(i, j)$ is the product of the frequencies of the responding genotype of each locus, meaning $\mathbf{P}(i, j) = f_1(i) f_2(j)$. For a two-locus disease model, we have

$$\begin{cases} b\sum_{i=1}^{3}\sum_{j=1}^{3}\mathbf{R}(i, j)\mathbf{P}(i, j) = p(D) \\ \dfrac{p(D \mid Aa) / p(D \mid AA)}{\left(1-p(D \mid Aa)\right) / \left(1-p(D \mid AA)\right)} - 1 = \lambda \end{cases}$$

which is transformed to

$$\begin{cases} b\sum_{i=1}^{3}\sum_{j=1}^{3}\left(\mathbf{R}(i, j) f_1(i) f_2(j)\right) = p(D) \\ \dfrac{\sum_{j=1}^{3}\left(\mathbf{R}(2, j) f_2(j)\right) / \sum_{j=1}^{3}\left(\mathbf{R}(1, j) f_2(j)\right)}{\sum_{j=1}^{3}\left(\left(1-\mathbf{R}(2, j)b\right) f_2(j)\right) / \sum_{j=1}^{3}\left(\left(1-\mathbf{R}(1, j)b\right) f_2(j)\right)} - 1 = \lambda \end{cases}$$

Given the marginal effect size λ and the population prevalence $p(D)$, the solution of two model parameters (*b* and *f* from **R**) can easily be obtained.

In simulation studies, we did not include the genotypes of the causative loci in the genotype-phenotype data. Instead, the genotypes of markers that are in LD with the causative loci were recorded in the data. We code the minor allele as 0 and the common allele as 1 here. Given p_0 as the MAF for a disease causative locus allele A_c, q_0 as the MAF of the corresponding marker allele A_m, and the LD measured by r^2, the target here is to calculate the conditional probability $\left\{Q_{i,j} = p(A_m = j \mid A_c = i)\right\}$. We have the equations as follows:

$$\begin{cases} p_0 Q_{0,0} + (1-p_0)Q_{1,0} = q_0 \\ \dfrac{(p_0 Q_{0,0}(1-p_0)(1-Q_{1,0}) - p_0(1-Q_{0,0})(1-p_0)Q_{1,0})^2}{p_0(1-p_0)q_0(1-q_0)} = r^2 \end{cases}$$

Since there are two equations for two variables, $Q_{0,0}$ and $Q_{1,0}$, we can easily solve the equations to obtain $Q_{0,0}$ and $Q_{1,0}$.

Assuming Hardy-Weinberg equilibrium, the transition probability matrix for marker genotypes given causative locus genotype is as follows:

$$\mathbf{P} = \begin{pmatrix} Q_{0,0}{}^2 & 2Q_{0,0}(1-Q_{0,0}) & (1-Q_{0,0})^2 \\ Q_{0,0}Q_{1,0} & Q_{0,0}(1-Q_{1,0}) + (1-Q_{0,0})Q_{1,0} & (1-Q_{0,0})(1-Q_{1,0}) \\ Q_{1,0}{}^2 & 2Q_{1,0}(1-Q_{0,0}) & (1-Q_{1,0})^2 \end{pmatrix}.$$

Number of irrelevant SNPs

If we test all possible combinations of *n* SNPs in the observed data and adjust the *p*-value with an exact correction method (e.g., Bonferroni correction), the probability of type I error could be controlled at the predefined level (0.05 in the paper), under the condition that the test statistic (e.g., the Chi-squared statistic) has good asymptotic characteristics. Consequently, the type I errors of all the four methods compared in main text could be expected to be controlled at the predefined level (0.05).

To validate this, we provide Figure S1 that gives the result of the average numbers of irrelevant SNPs identified by the four methods in our simulation studies. In the figure, the average number of irrelevant SNPs for a parameter setting (sub-model) is calculated by counting the number of unassociated SNPs being identified at the significance level 0.05 after Bonferroni correction and dividing this number by the total number of simulated datasets for the parameter setting (i.e., 100). The results for *epi*MODE are obtained using the selection-testing-correction method. The cutoff value of the posterior probability for selecting epistatic modules for further statistical test is 0.20. Note that, this cutoff value may influence the number of modules that goes to the statistical test procedure, but will not influence the inclusion of irrelevant SNPs in identified epistatic modules much because our Bonferroni correction is based on the total number of SNPs instead of the number of selected SNPs. We can see that in most cases, the average number of irrelevant SNPs for *epi*MODE, BEAM and single-locus Chi-squared test are all controlled at about 0.05, while stepwise logistic regression generally identified more irrelevant SNPs.

Impact of Dirichlet hyper-parameters

In order to investigate the influence of Dirichlet hyper-parameters in the proposed method, we have also tried 0.3, 0.7, and 1.0 besides 0.5 that is used in the main text. The results are shown in Figure S2. Basically, as we can see from the results, the values of Dirichlet hyper-parameters do

not influence the performance of the method much. We also notice that when the MAF is small (e.g., 0.05), smaller Dirichlet hyper-parameters tend to yield higher powers. This observation might be due to the fact that the frequencies of genotypes of a locus (or combinatory genotypes of several loci) tend to be either large or small when MAFs are small, because of the Hardy-Weinberg equilibrium. Consequently, smaller Dirichlet hyper-parameters might fit this situation better than a flatter prior distribution. On the other hand, when MAF is large, larger Dirichlet hyper-parameters tend to have higher powers. This observation might be due to the fact that the frequencies of genotypes of a locus (or combinatory genotypes of several loci) could be more flexible when MAFs are larger, and thus a flatter hyper-parameter may fit the model better.

Estimation of the penetrance of the combination of rs1394608 and rs3743175 in AMD

The penetrance of the combination of rs1394608 and rs3743175 is estimated in the following Bayesian formula

$$p(D \mid G_{LC}(i,j)) = \frac{p(G_{LC}(i,j) \mid D)\, p(D)}{p(G_{LC}(i,j))},$$

where $G_{LC}(i,j)$ is a genotype of the locus combination with the first locus being its *i*-th genotype and the second locus being its *j*-th genotype, $i,j=1,2,3$. $p(D)$ is the prevalence of the disease. $p(G_{LC}(i,j))$, the distribution of the genotype in the whole population where the disease status is not considered, is estimated with the distribution in control. However, as a result of insufficient sample size, it is not proper to use the distribution of genotypes in the sampled control data set instead of $p\left(G_{LC}(i,j)\right)$. We use the least square estimates to infer $p\left(G_{LC}(i,j)\right)$. We assume the minor allele frequencies of the two SNPs as MAF_1 and MAF_2, respectively. Assuming Hardy-Weinberg equilibrium, the genotype frequencies for the locus L_k is $f_k = \left((1-MAF_k)^2, 2MAF_k(1-MAF_k), MAF_k{}^2\right)$ for the homozygosity for common allele, the heterozygosity and the homozygosity for less allele, respectively. Assuming independent distribution of the two SNPs, the frequency for a combinatory genotype $p(G_{LC})$ is the product of the frequencies of the two genotypes that make the combinatory genotype, which means $p(G_{LC}(i,j)) = f_1(i) f_2(j)$. The estimate of $p(G_{LC}(i,j))$ is now transferred into an optimization problem as:

$$\arg\min_{MAF_1, MAF_2} \sum_{i=1}^{3}\sum_{j=1}^{3}\left[p\left(G_{LC}(i,j)\right) - p\left(G_{LC}(i,j) \mid U\right)\right]^2$$

where $p(G_{LC}(i,j) \mid U)$ is the frequency for combinatory genotype $G_{LC}(i,j)$ in control. After the optimized MAF_1 and MAF_2 are obtained, we can get $p\left(G_{LC}(i,j)\right)$ and then the estimate of $p\left(D \mid G_{LC}(i,j)\right)$, $i,j=1,2,3$.

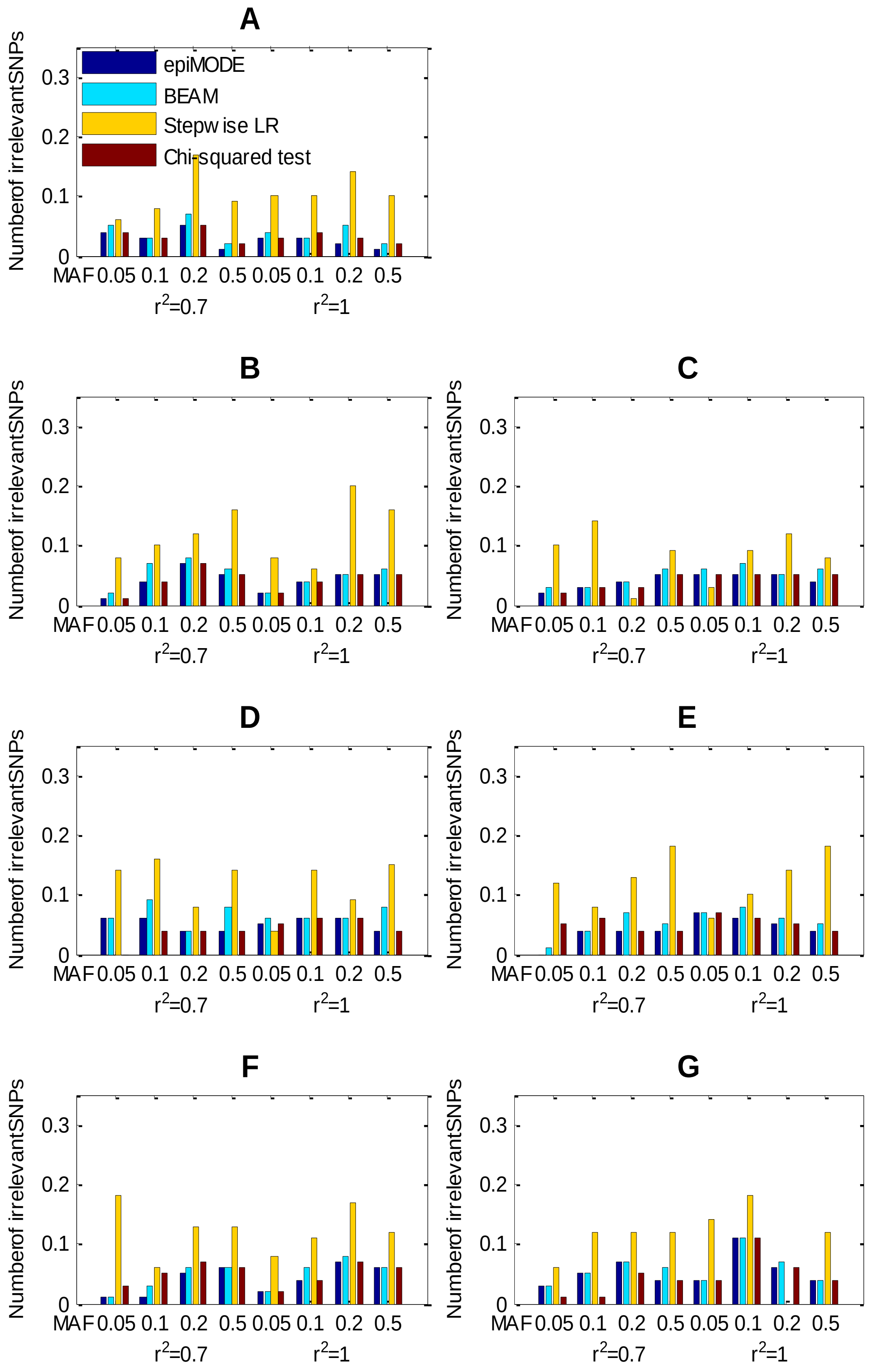

Figure S1. Average numbers of irrelevant SNPs.

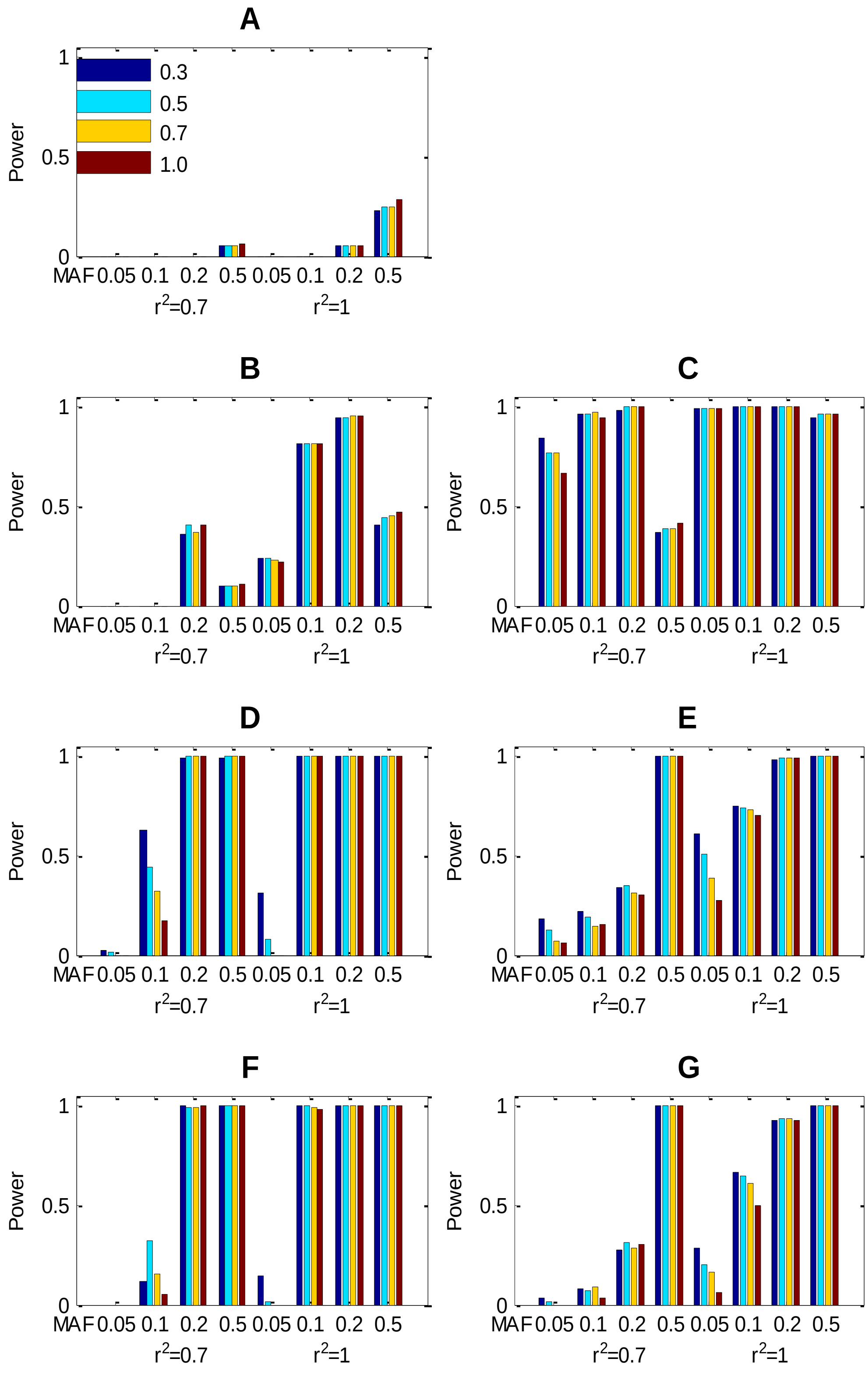

Figure S2. Performance of epiMODE with different Dirichlet hyper-parameters.

References

Zhang, Y. and J. S. Liu (2007). "Bayesian inference of epistatic interactions in case-control studies." Nat Genet **39**(9): 1167-1173.
